# Construction of a High-Density Genetic Map of *Acca sellowiana* (Berg.) Burret, an Outcrossing Species, Based on Two Connected Mapping Populations

**DOI:** 10.3389/fpls.2021.626811

**Published:** 2021-02-23

**Authors:** Marianella Quezada, Rodrigo Rampazo Amadeu, Beatriz Vignale, Danilo Cabrera, Clara Pritsch, Antonio Augusto Franco Garcia

**Affiliations:** ^1^Laboratorio de Biotecnología, Departamento de Biología Vegetal, Facultad de Agronomía, Universidad de la República, Montevideo, Uruguay; ^2^Laboratório de Genética Estatística, Departamento de Genética, Escola Superior de Agricultura “Luiz de Queiroz”, Universidade de São Paulo, Piracicaba, Brazil; ^3^Mejoramiento Genético, Departamento de Producción Vegetal, Estación Experimental de la Facultad de Agronomía, Universidad de la República, Salto, Uruguay; ^4^Programa de Investigación en Producción Fruticola, Instituto Nacional de Investigación Agropecuaria (INIA), Estación Experimental “Wilson Ferreira Aldunate”, Canelones, Uruguay

**Keywords:** *Acca sellowiana*, feijoa, pineapple guava, genotyping by sequencing, composite genetic map, multiparent family, Myrtaceae

## Abstract

*Acca sellowiana*, known as feijoa or pineapple guava, is a diploid, (2*n* = 2*x* = 22) outcrossing fruit tree species native to Uruguay and Brazil. The species stands out for its highly aromatic fruits, with nutraceutical and therapeutic value. Despite its promising agronomical value, genetic studies on this species are limited. Linkage genetic maps are valuable tools for genetic and genomic studies, and constitute essential tools in breeding programs to support the development of molecular breeding strategies. A high-density composite genetic linkage map of *A. sellowiana* was constructed using two genetically connected populations: H5 (TCO × BR, *N* = 160) and H6 (TCO × DP, *N* = 184). Genotyping by sequencing (GBS) approach was successfully applied for developing single nucleotide polymorphism (SNP) markers. A total of 4,921 SNP markers were identified using the reference genome of the closely related species *Eucalyptus grandis*, whereas other 4,656 SNPs were discovered using a *de novo* pipeline. The individual H5 and H6 maps comprised 1,236 and 1,302 markers distributed over the expected 11 linkage groups, respectively. These two maps spanned a map length of 1,593 and 1,572 cM, with an average inter-marker distance of 1.29 and 1.21 cM, respectively. A large proportion of markers were common to both maps and showed a high degree of collinearity. The composite map consisted of 1,897 SNPs markers with a total map length of 1,314 cM and an average inter-marker distance of 0.69. A novel approach for the construction of composite maps where the meiosis information of individuals of two connected populations is captured in a single estimator is described. A high-density, accurate composite map based on a consensus ordering of markers provides a valuable contribution for future genetic research and breeding efforts in *A. sellowiana*. A novel mapping approach based on an estimation of multipopulation recombination fraction described here may be applied in the construction of dense composite genetic maps for any other outcrossing diploid species.

## 1. Introduction

*Acca sellowiana* (Berg.) Burret, commonly known as feijoa or pineapple guava, is an agronomically promising fruit tree species, native to Uruguay and southern Brazil. This diploid (2*n* = 2*x* = 22) outcrossing species presents a small genome (245 Mb) (da Costa et al., 2008) and the basic haploid number of *n* = 11, largely conserved in the Myrtaceae family (Grattapaglia et al., 2012). The species stands out in the novel market of health-promoting and functional food due to the nutraceutical value of its fruits, rich in vitamin C (28 mg/100 g), hydrocarbons, minerals, iodine (3 mg/100 g), and bioflavonoids (Weston, 2010). However, feijoa can still be considered a minor crop due to its seasonal presence on the market and a small cultivation area worldwide. The development of new cultivars with superior fruit quality traits and adapted for new environments is needed to accelerate the commercial exploitation of the species. For fruit tree species, like feijoa, the breeding process is slow and costly because of the long juvenile period, extensive phenotyping cost, and limited field space (Byrne, 2012). For this reason, the development of genetic and genomic resources, such as genetic maps, represents a suitable strategy to overcome these limitations and speed up the breeding progress.

As a minor crop, genomic resources in *A. sellowiana* are limited and not yet useful in breeding programs. Few studies document the molecular diversity of the species using low-throughput molecular markers (Dettori and Palombi, 2000; dos Santos et al., 2007; Pasquariello et al., 2015; Donazzolo et al., 2020; Saifert et al., 2020) and a limited number of molecular markers have been specifically designed for *A. sellowiana* (dos Santos et al., 2008; Klabunde et al., 2014). Amplified fragment length polymorphisms (AFLP), intersimple sequence repeat (ISSR), and a small number of simple sequence repeats (SSR) markers were used in the construction on the first genetic map of the species (Quezada et al., 2014). This map along with those developed for the species *Psidium guajava* (Padmakar et al., 2015) represent the only ones developed for fruit species within the Myrtaceae family. With the advance of new sequencing technologies, single nucleotide polymorphisms (SNPs) have become the most useful type of marker for genetic analysis. The next-generation sequencing (NGS) technology coupled with enzyme-based complexity reduction and DNA barcoding have been used to simultaneously discover and genotype a large number of SNPs in a single experiment (Baird et al., 2008; Elshire et al., 2011). This low cost strategy, implemented in protocols such as genotyping by sequencing (GBS) (Elshire et al., 2011), provides a fast, efficient, and cost-effective strategy to obtain a significant number of markers. It represent an invaluable asset for minor or underutilized crops, such as *A. sellowiana*, with relatively few genomic and genetic resources. Using SNPs from GBS, high-density linkage maps for many commercial fruit tree species (Barba et al., 2014; Gardner et al., 2014; Bielenberg et al., 2015) as well as for minor crops have been developed (Ward et al., 2013; Covarrubias-Pazaran et al., 2016).

High-density genetic maps are extremely valuable tools to investigate the composition and organization of genomes for comparative genetic mapping analysis, chromosome-based genome assembly, physical and genetic map integration, and candidate gene/QTL cloning (Bartholomé et al., 2014; Mathew et al., 2014; Fierst, 2015; Guajardo et al., 2015; Li et al., 2017; Jaganathan et al., 2020). Genetic maps are also useful tools in breeding programs, fundamentally to establish associations between molecular markers and agronomical traits, providing the basis for future strategies of QTL identification and marker-assisted selection (MAS) (Troggio et al., 2012). For outcrossing species, genetic maps are typically based on single full-sib mapping populations derived from crosses of two highly heterozygous parents. The pseudo-test cross strategy was first developed to construct genetic maps in these populations. In this strategy, markers heterozygous only in one parent, therefore segregating in a 1:1 ratio, are used to generate two separate individual linkage maps (Grattapaglia and Sederoff, 1994). Later, methods using information from all markers simultaneously (considering dominant and codominant markers, heterozygous either in one or both parents, with segregation ratios 1:1:1:1, 3:1, 1:2:1, and 1:1) were developed to construct integrated genetic linkage maps (Maliepaard et al., 1997; Wu et al., 2002a,b). The OneMap R (Margarido et al., 2007) package implements the maximum likelihood approach to simultaneously estimate linkage and linkage phases between markers; in addition, it provides a multipoint estimation for the recombination fraction using a hidden Markov model (HMM) (Wu et al., 2002b), which is a reliable procedure (Mollinari et al., 2009). This approach was successfully applied in the construction of integrated genetic maps for several outcrossing species (Palhares et al., 2012; Pereira et al., 2013; Quezada et al., 2014; Balsalobre et al., 2017).

The construction of accurate genetic maps faces two main difficulties. First, it is necessary to have a high number of polymorphic markers to obtain a comprehensive coverage of the genome. Second, large mapping populations need to be developed to accurately estimate genetic distances. Presently, developing a large number of markers is not a limiting factor, even for species with few genetic and genomic resources (Davey et al., 2011). However, most of the mapping populations of fruit trees that have been developed in breeding programs have a small to medium progeny size because of the high cost of maintaining these populations and the limited orchard space (Peace and Norelli, 2009). Capitalizing on existing breeding-mapping populations provides an opportunity to develop genetic maps including information of multiple populations, and to overcome the limitations of a reduced number of recombination events captured in single mapping populations. The integration of single-population genetic maps into a composite map improves the accuracy and resolution of maps by correcting the order and position of markers, increasing genome coverage, and filling out genomic regions lacking polymorphic markers in specific crosses (Khan et al., 2012; Pootakham et al., 2015). Moreover, composite genetic maps enable a more precise estimation of QTL effects and positions (Di Pierro et al., 2016) as well as facilitates the transfer of marker information and genetic predictions between populations (Kuhn et al., 2017). Many strategies have been proposed to construct consensus composite maps applied to outcrossing species (Khan et al., 2012; Pootakham et al., 2015; Bodénès et al., 2016; Schlautman et al., 2017). In some cases, the recombination fraction is estimated combining all genotypic data sets from multiple populations (de Givry et al., 2005; Van Ooijen, 2006), whereas in others, map integration is achieved based on the marker order and distances from individual maps (Wu et al., 2011; Endelman and Plomion, 2014). The inability to solve inconsistencies or ordering conflicts between maps as well as inflated genetic distances can be considered the main limitations in both approaches (Khan et al., 2012).

The objective of this study was to develop a reference and composite linkage genetic map for *A. sellowiana*, integrating the genetic information from two connected *F*_1_ populations. For this purpose, two mapping populations segregating for fruit quality traits with the same female parent were used. For the first time in the species, a high-throughput GBS approach was successfully applied to identify SNP markers distributed throughout the genome. A new statistical model was implemented into OneMap (Margarido et al., 2007) and used to build a composite map using information from multiparental outcrossing populations. The two saturated individual genetic maps and the composite one provide a framework for future genetic and genomic studies, and they will be useful for future studies of marker-trait association and QTL mapping, which in turn will speed up the breeding process in *A. sellowiana*.

## 2. Materials and Methods

### 2.1. Mapping Populations

Two full-sib *F*_1_ populations genetically connected by a common parental genotype and segregating for relevant fruit quality traits were used to construct an integrated composite map. These populations were generated by the crossing of non-inbred clones TCO × BR (H5 population) and TCO × DP (H6 population), and consist of 160 and 184 genotypes, respectively. TCO was used as female parent in both crosses. The three parental genotypes were chosen on the basis of their agronomic features: TCO, collected from the wild, has small fruits with tasty and smooth pulp, as well as a thin light-green skin; BR, collected from a commercial orchard, has large fruits with tasteless pulp and a rough, thick, dark-green skin; DP, collected from a commercial orchard has a high yield of medium size fruits, and a rough, medium-thick, dark-green skin. The mapping populations were developed in 2008 by the Native Fruits Breeding Program (Universidad de la República (UdelaR) and Instituto Nacional de Investigación Agropecuaria (INIA), Uruguay). H5 population was planted in the Experimental Station Salto of Facultad de Agronomía-UdelaR, Uruguay (31°19′ S, 57°41′ W), whereas H6 population was planted in the Experimental Station Salto Grande of INIA, Uruguay (31°25′ S, 57°37′ W).

### 2.2. DNA Extraction and Library Construction

Leaf material was collected from a total of 344 individuals, and maintained in paper bags with silica gel at −20° until DNA extraction. Genomic DNA was extracted using DNeasy Plant Kit (Qiagen, Germantown, MD, USA) according to the manufacturer's protocol. DNA quality was checked by electrophoresis on 0.8% agarose gel and quantified by NanoDrop ND-1000 spectrophotometer (NanoDrop Technologies, Wilmington, DE, USA). GBS libraries were prepared according to the original GBS protocol (Elshire et al., 2011) in the Institute for Genomic Diversity (Cornell University, Ithaca, NY, USA). For optimization of the GBS protocol, three different restriction enzymes were compared. Test libraries were prepared separately with the five-cutter *ApeK*I, and six-cutter *Eco*T22I and *Pst*I. The fragment size distribution for each test library was evaluated using an Experion system (Bio-Rad, Hercules, CA, USA). Both six-cutter enzymes produced the smallest fragment pool (adequate for higher SNP sequencing coverage), but the *Eco*T22I library contained a detectable amount of repetitive DNA. Thus, the library derived from *Pst*I was selected as it comprised the largest number of fragments minor to 500 bp, and presented a smooth profile for fragment size distribution indicative of very low content of repetitive DNA. DNA samples and blank negative controls were prepared in two 192-plex libraries and were sequenced (single-end reads of 100 bp in length) twice in a HiSeq2000 sequencer (Illumina R Inc., San Diego, CA, USA). To guarantee a satisfactory coverage and confidence in calling heterozygous genotypes, the three parental genotypes had 12 samples each, and all the samples reached a coverage equivalent to 96-plex.

### 2.3. SNP and Genotype Calling

The quality of the raw sequence (per base sequence quality, average read quality score, per base N content) was evaluated using the FastQC v0.11.5 (http://www.bioinformatics.babraham.ac.uk/projects/fastqc). For SNP calling, two strategies implemented in the Tassel GBS software were employed comprising the Discovery pipeline using a reference genome (Tassel v4.0) as well as the UNEAK network pipeline (Tassel v3.0) (Lu et al., 2013; Glaubitz et al., 2014).

Raw sequences were filtered, discarding reads lacking the barcode, not having the expected *Pst*I cut-site, or containing uncalled bases (i.e., Ns) within the first 64 bp subsequent to the barcode. The barcodes was removed in the raw sequences and the remainder of the sequences trimmed to 64 bases. Filtered reads were grouped in tags, and only those with a minimum coverage depth of 5 were retained (parameter -c 5 at the MergeTaxaTagCountPlugin step). For the Discovery pipeline, tags were aligned to the *E. grandis* reference genome (*E. grandis* v1.0, JGI, http://www.phytozome.net) using Bowtie v1.2.0 (Langmead and Salzberg, 2012) with default parameters. The *E. grandis* genome stands out as the reference genome for the Myrtaceae family (Grattapaglia et al., 2012).

In the UNEAK strategy, tags were aligned to each other and only a single base-pair mismatch was considered. Due to the presence of repeated or paralogous sequences in the genome, many tag networks are generated. These networks were filtered (error tolerance rate set at 0.03) and only reciprocal tags were maintained representing potential SNPs. Discovery and UNEAK data were exported as *vcf* files (information-rich variant call format) where the read depth of the reference and alternative allele is saved (Danecek et al., 2011). SNP markers identified by the Discovery strategy were denoted as “Eg” followed by the chromosome number and the position (bp) on the *E. grandis* reference genome. SNPs identified using the UNEAK pipeline were named as “Un1” plus a number referring to the tags network. Redundant markers found in both Discovery and UNEAK pipelines were removed from UNEAK data set, and assigned both Discovery and UNEAK identification (e.g., *Eg8_53239405:*Un1_5092215). Consequently, when total number of mapped markers is reported, redundant markers are counted once.

A quantitative genotype calling was performed for each population separately using SuperMASSA software (Serang et al., 2012). For genotype calling, this software considers the expected allele distribution in an *F*_1_ population, as well as the relative site coverage (read counts) of each allele (Garcia et al., 2013). In a previous step, markers were filtered out for quality; only biallelic markers with less than 25% of missing data were retained. The *F*_1_ segregation model with diploid level was fitted in SuperMASSA, where each of the 12 replicates of parental genotypes were analyzed separately, to provide additional constraints during estimation (Pereira et al., 2018). Following the recommendations of (Mollinari and Serang, 2015), SuperMASSA naive posterior report threshold was set to zero and individual posterior probability values were estimated. The median of all individual posterior probabilities was used as a quality control, so only SNPs with posterior values higher than 0.8 were selected. The SuperMASSA *vcf* file output was formatted for mapping purposes using vcfR (Knaus and Grünwald, 2017) and OneMap (Margarido et al., 2007) R packages (R Development Core Team, 2017), following the instructions given in the OneMap software tutorial (http://augustogarcia.me/onemap/vignettes_highres/Outcrossing_Populations.html#importing-data). Following the notation of (Wu et al., 2002a), markers were classified into four groups (“A-D”) according to their respective cross type. For GBS-SNP markers in an *F*_1_ population, only three marker cross types are informative for genetic map construction: “B3.7” (“ab” × “ab”), “D1.10” (“ab” × “aa”), and “D2.15” (“aa” × “ab”). The “D” group comprises markers segregating in a 1:1 ratio, i.e., heterozygous in only one parent, also called testcross markers. The “B3.7” markers are heterozygous and symmetric in both parents, with an expected 1:2:1 segregation ratio. In addition, for markers segregating on both populations, five new configuration types were defined, considering the simultaneous segregation pattern. If the common maternal genotype is heterozygous for a locus (“ab”), the paternal genotype could be heterozygous in both populations (“ab” × “ab”/“ab” × “ab”, “B3.7-B3.7”), only heterozygous in H5 population (“ab” × “ab”/“ab” × “aa,” “B3.7”-“D1.10”), only heterozygous in H6 population (“ab” × “aa”/“ab” × “ab,” “D1.10”-“B3.7”) or homozygous in both populations (“ab” × “aa”/“ab” × “aa,” “D1.10”-“D1.10”). For markers homozygous in the female parent, the only possible configuration is that the paternal genotypes were heterozygous in both populations (“aa” × “ab”/“aa” × “ab,” “D2.15”-“D2.15”), or otherwise there is no information to integrate the populations.

### 2.4. Construction of Maps for Individual Populations

For each population, a data set including the SNPs identified by Discovery and UNEAK pipelines was generated. For H5 population, 493 previously identified markers (ISSR, AFLP, and SSR) were also included (Quezada et al., 2014). Integrated genetic maps for H5 and H6 populations were constructed using OneMap (Margarido et al., 2007) R package (current version available at https://github.com/augusto-garcia/onemap). This software implement the method proposed by Wu et al. (2002a,b) for simultaneous multipoint estimation of the recombination fraction and linkage phases between markers. Using a multipoint approach, that uses information from multiple markers to estimate the recombination fractions, the most adjacent informative markers fill the lack of information in markers with missing data. Considering that both populations have a comparable number of individuals and genotyped markers, the same procedure was applied to construct both maps.

First, the segregation of each marker was evaluated for goodness-of-fit to the expected Mendelian segregation ratio by a chi-square test, followed by a Bonferroni correction for multiple testing considering 0.05 the overall significance level. Two-point linkage analysis was carried out between all pairs of markers using the rf_2pts command. Linkage groups (LGs) were established with a logarithm of the odds (LOD) score of 7.5 (estimated value based on the number of two-point tests and multiple test correction) and a maximum recombination fraction of 0.35. Recombination frequency was converted to genetic map distances (cM) using the Kosambi function (Kosambi, 1944). To order groups, the order_seq function was used; this selects a subset of informative markers (6–7 markers), wherein the remaining markers were introduced. A framework map was evaluated, and markers exhibiting a suspect position (ordering problems), producing gaps at the ends of the groups or inflating LGs size were removed. Once the framework map presents a reliable initial order, the try_seq algorithm (Lander and Green, 1987) was used to integrate previously removed markers and also markers that remained unmapped. Incorrect allocation of markers was visually inspected using a heatmap plot (graphical representation of the recombination fraction and LOD score between markers). Considering that the GBS-SNP markers could have genotyping errors, a probability of 0.05 (default = 0.01) of error was considered to construct the genetic maps. The ripple algorithm was used to verify alternative local orders (Lander and Green, 1987). LGs were numbered according to the chromosome number of *E. grandis* based on the location of markers identified using this genome as reference. Finally, the linkage maps were drawn using the MapChart v2.3 software (Voorrips, 2002).

### 2.5. Construction of a Composite Integrated Genetic Map

A new approach to construct a composite genetic map using the information of the two connected populations was developed. The proposed model assumes a homogeneous recombination fraction between populations, which is estimated using the segregation data for both populations simultaneously. Therefore, this method will have a higher statistical power, resulting in more accurate estimates of markers distances and order.

To build a composite genetic map, H5 and H6 individual integrated genetic maps were compared to identify common markers. These markers were identified by name, considering that SNP calling was performed on the whole data set. For these markers, the location in the same LG as well as the marker order was registered in both H5 and H6 maps. A new data set for each LG was created, containing information only of the shared markers presented in homologous LGs.

The two-point recombination fraction between common markers in each LG was estimated using the segregation data of the two populations simultaneously. The maximum likelihood estimator of the recombination fractions and LOD score formulas were adapted from Maliepaard et al. (1997), only for the marker configurations present in the data. To estimate recombination fractions in *F*_1_ populations, estimates of linkage phase between markers is necessary. Considering two consecutive markers in a homologous chromosome, the alleles of a pair of loci can be present at coupling (C) or repulsion (R) phase configuration. Because in our populations the same female parental genotype is shared, the linkage phase of markers on this parent was equal in both crosses. Thus, considering simultaneously the crosses “TCO × BR” and “TCO × DP,” eight linkage phase assignments were evaluated (*f* and *m* indicated female and male parent, respectively): (i) C*f*C*m*-C*f*C*m*, (ii) C*f*C*m*-C*f*R*m*, (iii) C*f*R*m*-C*f*C*m*, (iv) C*f*R*m*-C*f*R*m*, (v) R*f*C*m*-R*f*C*m*, (vi) R*f*C*m*-R*f*R*m*, (vii) R*f*R*m*-R*f*C*m*, and (viii) R*f*R*m*-R*f*R*m*. An illustration of the recombination fraction estimation between markers with different configuration type is provided in Supplementary Data Sheet 1.

A multipoint approach was implemented using an HMM (Lander and Green, 1987) with the expectation maximization (EM) algorithm (Dempster et al., 1977). To estimate the recombination fraction, and due to the meiosis in one population is independent to the meiosis of the another population, the expectation step was computed on each population separately and the maximization step was performed considering segregation information for both populations simultaneously. The new R function rf_2pops implement this (Supplementary Data Sheet 1).

To implement the multipoint approach, the marker order of the individual H5 and H6 maps was tested. The marker order with the highest likelihood was selected as initial order of the composite map. Then, all the markers with ordering conflict between the individual maps were evaluated in all possible positions on the composite map and the position with highest likelihood was selected. A final local order verification was performed with ripple algorithm implemented in the ripple_2pops command. The final marker order of the composite map was used to individual H5 and H6 maps to facilitate comparisons.

Finally, the markers present only in H5 or H6 individual maps were incorporated to the composite map. For these markers, we only had recombination information from one population, so it was not possible to estimate a multipopulation recombination fraction. To include these unique markers, genetic distances in cM were re-estimated by multiplying by a specific scaling factor. In the case of unique markers between consecutive common markers, the scaling factor was calculated by dividing the genetic distance in the composite map by the distance in the individual map for the same interval. For markers at the end of LGs, the total map length of the composite map divided by the total map length of individual map for each LG was used.

To perform the analysis, new functions of the OneMap package were developed to handle information for multiple populations. The functions developed in this work (rf_2pops and ripple_2pops) alongside with a user-friendly tutorial (Supplementary Data Sheet 2) are available at https://github.com/augusto-garcia/onemap2pop. They will be integrated into OneMap package in a near future.

## 3. Results

### 3.1. GBS Libraries

A total of 859,454,459 reads of 100 bp length were obtained from the complete sequencing of the two libraries. Read number and sequence quality were shown to be comparable for the two libraries and the two sequencing processes using FastQC. Read quality was similarly good in both libraries, with an average base quality score higher than 30 (99.9% base call accuracy). The total number of reads per progeny ranged from 60 thousand to 6.2 M. The 12 fold-repeated samples of parental genotypes resulted in a deeper coverage, with a 3.2 M average number of reads for the parental genotypes and 2.2 M per progeny. Two samples (H5_095 and H6_156) that presented a relatively low numbers of reads (<150,000), representing less of the 10% of the mean reads per sample of the lane on which they were sequenced, were removed for subsequent analysis.

### 3.2. SNP Calling

**Figure 1:**
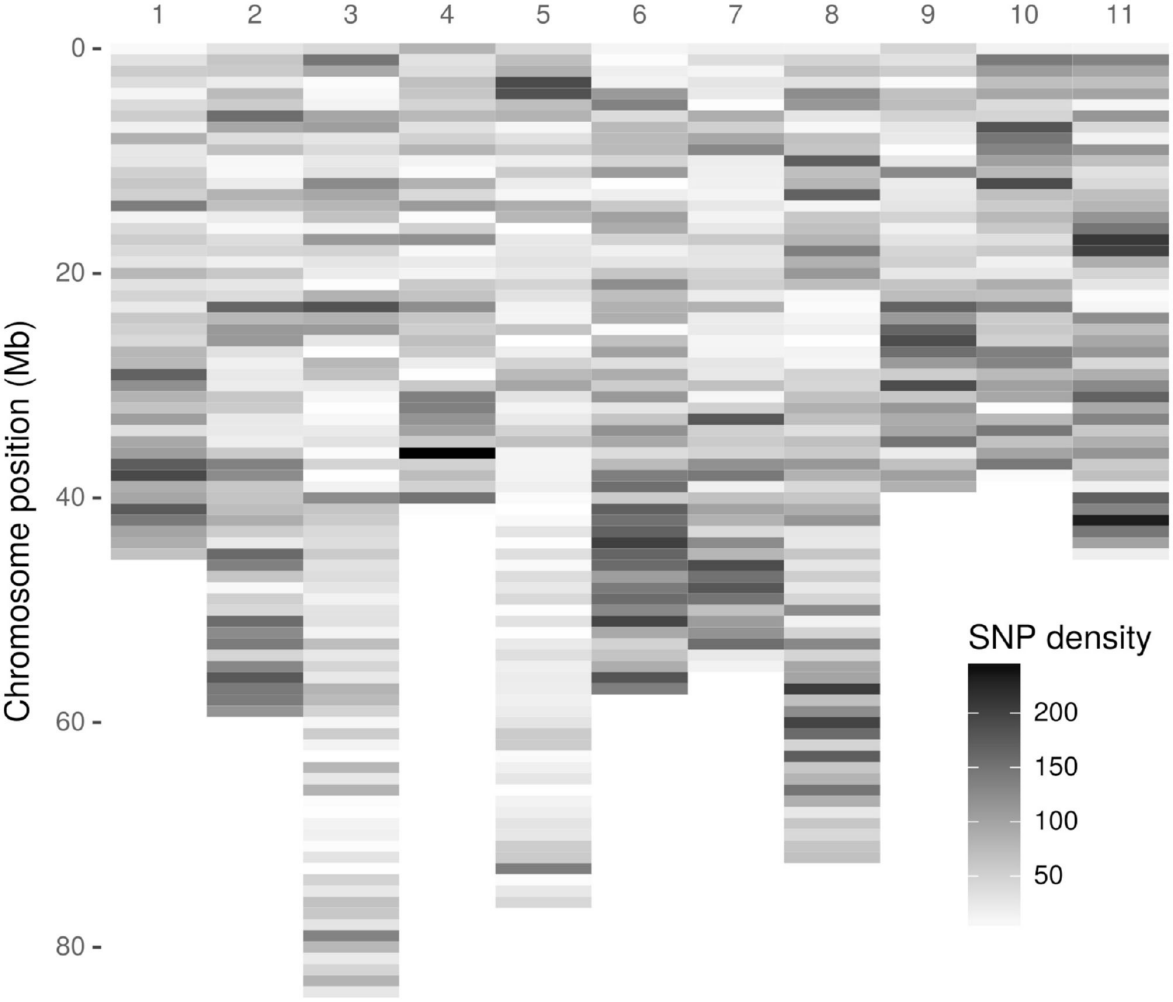
Density distribution of Discovery-identified *Acca sellowiana* single nucleotide polymorphisms (SNPs) across the *Eucalyptus grandis* chromosomes. The SNP density was estimated in a 1 Mb window. The y-axis indicates the physical position in Mb. Of the 43,377 Discovery-identified SNPs, 41,815 were distributed across the *E. grandis* chromosomes and showed in the graphic. The remaining 1525, identified in sequence scaffolds, are not displayed.

As a first step, 3,051,475 high-quality tags (unique sequence from one or more high-quality reads) were identified. These GBS tags were aligned to the *E. grandis* reference genome in the Discovery pipeline. Of these, 643,863 (21.10%) aligned exactly once in the reference genome and 280,937 (9.21%) aligned in multiple positions, resulting in an overall alignment rate of 30.31% (924,800 tags). As a result of the Discovery approach, 43,377 putative SNPs were identified. Figure 1 presents the SNP markers distribution across the *E. grandis* chromosomes. Using the UNEAK pipeline, 44,889 high-quality pairwise alignments were identified, of which 13,430 putative SNPs were obtained.

### 3.3. Genotype Calling

A total of 56,807 SNPs, comprising both Discovery and UNEAK SNP data sets, were available for genotype calling (Table 1). This number lowered to 53,051 (93.38%) after filtering out markers with no identified variation (missing alternative allele) or triallelic markers per genotype. After removing markers with more than 25% of missing data, a total of 27,595 (48.58%) and 25,670 (45.19%) SNPs were retained for H5 and H6 populations, respectively. For genotype calling, the filtered markers were subjected to quantitative genotyping following an *F*_1_ model implemented in SuperMASSA. Markers with the median of all individual posterior probabilities greater than 0.8 were selected. This criterion was chosen to ensure a high quality of genotypes as described by Garcia et al. (2013) and Mollinari and Serang (2015). Finally, a low number of overlapping markers between both Discovery and UNEAK SNP data sets was found for both H5 (174 SNPs) and H6 (88 markers) populations, and removed for the UNEAK data set. As a result, a set of 5350 (9.42%) and 4227 (7.44%) of high-quality SNPs was used for genetic mapping for H5 and H6 populations, respectively. Of the H5 population SNP set, 2875 (53.74%) markers were identified by the Discovery pipeline, so the remaining 2475 (42.26%) markers by the UNEAK pipeline. For the H6 population, 2046 (48.40%) markers and 2181 (51.60%) were identified using the Discovery and UNEAK approaches, respectively.

**Table 1:** Summary of single nucleotide polymorphism (SNP) marker number after filtering process for H5 and H6 populations.

	**Population H5**		**Population H6**	
**Pipeline**	**Discovery**	**UNEAK**	**Discovery**	**UNEAK**
Initial markers^*a*^	43,377	13,430	43,377	13,430
Triallelics^*b*^	39,650 (91.41%)	13,401 (99.78%)	39,650 (91.41%)	13,401 (99.78%)
25% missing data^*c*^	23,113 (53.28%)	4482 (33.37%)	21,395 (49.32%)	4275 (31.83%)
Probability genotype > 0.8^*d*^	2875 (6.63%)	2475 (18.43%)^*e*^	2046 (4.72%)	2181 (16.24%)^*e*^
Total	5350		4227	

a *Initial number of SNP markers identified simultaneously in both populations*.

b *Triallelics markers or with missing alternative allele were excluded*.

c *SNP markers with > 25% missing data were excluded*.

d *SNP markers with a median posterior probability > 0.8 from SuperMASSA software were selected*.

e *Redundant markers between Discovery and UNEAK sets were removed from UNEAK list*.

Considering the marker segregation patterns, SNP markers were classified into one of the three configuration marker types informative for genetic map construction: “B3.7” (“ab” × “ab”); “D1.10” (“ab” × “aa”); and “D2.15” (“aa” × “ab”) (Wu et al., 2002a). Of the 5350 segregating markers identified in H5 population, 1520 (28.41%), 1752 (32.75%), and 2088 (39.03%) were classified as “B3.7,” “D1.10,” and “D2.15,” respectively. For H6 population, a set of 4227 markers were identified, and of those: 993 (23.49%), 1520 (35.96%), and 1714 (40.55%) were “B3.7,” “D1,” and “D2,” respectively. Segregation distortion from the Mendelian expected ratios (1:2:1 for “B3.7” markers and 1:1 for “D1.10” and “D2.10” markers) was observed in 1263 (23.61%) markers of the H5 data set and 1112 (26.31%) markers of H6 data set.

**Figure 2:**
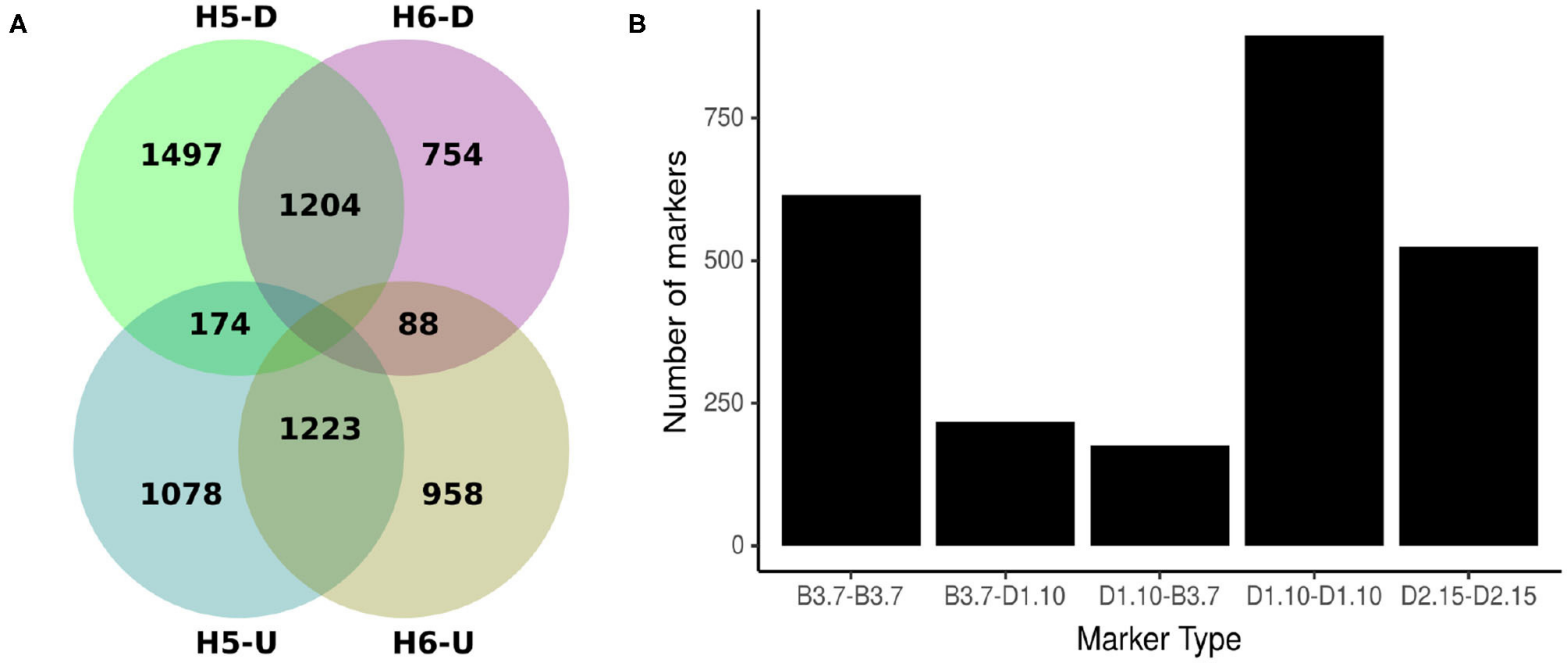
Origin and types of marker configuration for GBS-SNP markers identified for H5 and H6 populations. **(A)** Venn diagram showing common and unique single nucleotide polymorphisms (SNPs) between H5 and H6 populations identified by Discovery (D) and UNEAK (U) pipelines. **(B)** Distribution of five joint types of marker configurations for common markers between H5 and H6 populations. The joint marker configuration types considered the marker types in H5 and H6 populations simultaneously.

Comparing H5 and H6 SNP data sets, 2427 SNP were common. Of these shared markers, 1204 (49.61%) were identified by Discovery pipeline and 1223 (50.93%) by the UNEAK pipeline (Figure 2A). The segregation pattern of the common markers were inspected based on a joint marker configuration type. Markers with the same configuration type in both populations were the most frequent. For instance, the joint configuration D1.10-D1.10 accounted for the 36.88%, whereas the configuration B3.7-B3.7 and D2.15-D2.15 accounted for 25.34 and 21.59%, respectively. The B3.7-D1.10 and D1.10-B3.7 represented 8.94 and 7.25% of the configuration patterns for all the common markers, respectively (Figure 2B).

### 3.4. H5 and H6 Genetic Map

**Figure 3:**
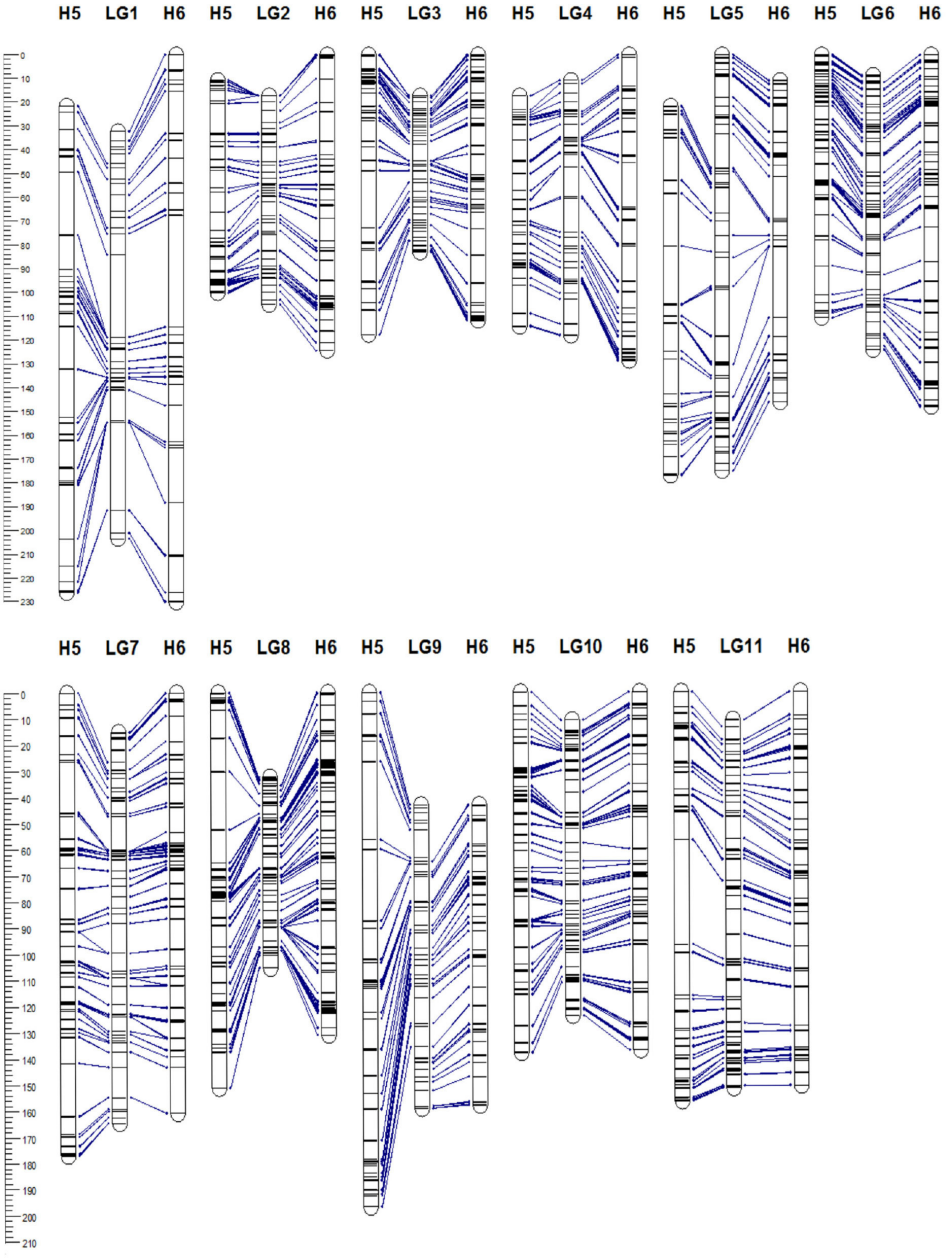
Genetic linkage map of *Acca sellowiana*. The composite linkage map (in the middle) integrated the information of the single population H5 (on the left) and H6 (on the right) maps. Map distances (cM) are indicated at the left side of the figure. Single nucleotide polymorphisms (SNP) markers common between linkage groups (LGs) of the three genetic maps are connected with lines.

The 5,350 segregating GBS-SNPs of H5 population were combined with 493 (100 ISSR, 386 AFLP, and 7 SSR) makers previously reported by (Quezada et al., 2014) for the same population. Therefore, 5,843 markers were combined for linkage analysis. The resultant H5 genetic map, derived from the “TCO × BR” cross, comprised 1,236 markers encompassing 11 LGs, corresponding to the haploid chromosome number of *A. sellowiana* (Table 2, Figure 3). The total length of the map was 1593 cM, with LG4 being the smallest group (97.14 cM) and LG1 the largest (204.43 cM). The number of markers per group ranged from 68 (LG5) to 164 (LG10) with a mean of 112.36 markers per LG.

**Table 2:** Description of H5, H6, and composite linkage maps for *A. sellowiana*.

	**H5 Map**			**H6 Map**			**Composite Map**		
**LG**	**No. of Markers**	**Length (cM)**	**Marker density**	**No. of Markers**	**Length (cM)**	**Marker density**	**No. of Markers**	**Length (cM)**	**Marker density**
LG1	92	204.43	2.22	87	230.28	2.65	131	171.17	1.31
LG2	84	89.40	1.06	92	119.69	1.30	123	87.89	0.71
LG3	105	117.80	1.12	128	111.49	0.87	163	65.92	0.40
LG4	91	97.14	1.06	106	128.43	1.21	125	107.30	0.86
LG5	68	155.31	2.28	74	135.35	1.83	131	174.94	1.34
LG6	136	110.69	0.81	149	147.86	0.99	200	115.46	0.58
LG7	134	176.24	1.31	137	160.69	1.17	194	149.43	0.77
LG8	143	151.17	1.06	172	130.98	0.76	251	73.00	0.29
LG9	106	196.89	1.86	106	114.94	1.08	158	116.00	0.73
LG10	164	137.70	0.84	134	136.94	1.02	235	112.95	0.48
LG11	113	156.56	1.38	117	150.71	1.29	186	140.83	0.76
Total	1236	1593.35	1.29	1302	1567.38	1.20	1897	1314.89	0.69

For the construction of the H6 genetic map, corresponding to the “TCO × DP” cross, 4227 GBS-SNPs were analyzed. A total of 1,302 markers were mapped in the H6 map, spanning the expected 11 LGs for the species (Table 2, Figure 3). The map covered a total distance of 1,567 cM, with the size of LGs ranging from 111.49 cM (LG3) to 230.28 cM (LG1). The average number of markers per LG was 118.36, with a minimum number of 74 markers in LG5 and a maximum of 172 markers in LG8.

Similar average distances between markers was achieved in both H5 and H6 genetic maps. The average marker densities were 1.29 and 1.20 cM for H5 and H6 map, respectively. The H5 map presented 15 gaps larger than 15 cM, whereas 10 were found in the H6 map. We also observed similarly low proportions of markers with segregation distortion in both H5 and H6 genetic maps. Thus, 3.80% (47 out of 1,256) and 3.69% (48 out of 1,302) of the mapped markers were distorted with a random distribution over the H5 and H6 map, respectively. In addition, both maps presented a similar distribution of the segregation patterns of the mapped markers. The D1 marker configuration type was the mostly mapped in both populations, with 711 (57.52%) and 705 (54,14%) markers in H5 and H6 genetic maps, respectively. H5 and H6 maps presented 423 (34.22%) and 522 (40.09%) D2 markers, respectively. The B3-type markers were the less frequently mapped, with 101 (8.17%) mapped markers in H5 maps and 75 (5.76%) in H6 map. Only one AFLP marker with C.8 configuration (“ao” × “ao”) was mapped in H5 map.

Of the 1,236 mapped markers in H5 genetic map, only 23 markers (12 AFLP, 5 ISSR, and 1 SSR) were previously mapped in the former H5 map (Quezada et al., 2014). The LG9 and LG11 presented the largest number of markers previously mapped, with 8 and 7 markers mapped in H5 former map. The reaming markers were mapped in the LG5, LG1, and LG3 (Supplementary Data Sheet 3).

### 3.5. Composite Genetic Map

In order to construct a composite map, 641 common markers between H5 and H6 map were identified. All these shared SNPs were located within the same LG in both maps, with a largely consistent overall order between LGs. The recombination fractions between these markers was re-estimated using the information of both populations simultaneously and the best order was achieved based on the likelihood. After constructing the composite map, 595 and 661, H5 and H6 unique markers respectively, were incorporated. Consequently, the composite map included a total of 1,897 markers along 11 LGs with a total length of 1,314 cM (Table 2, Figure 3). The average group length was 119.53 cM, with LG2 the smallest (65.92 cM) and LG5 the largest ones (174.94 cM). The number of markers per group ranged from 123 (LG2) to 251 (LG8) with a mean of 172.45. The average distance between markers was 0.69 cM, with only three gaps larger than 15 cM.

The 641 common markers in the composite map presented all five possible joint marker configuration types defined considering the segregation pattern in both populations simultaneously. However, markers heterozygous in the maternal genotype and homozygous in both paternal genotypes (“ab” × “aa”/“ab” × “aa,” “D1.10”-“D1.10”) were largely the most frequent, with 449 (70.05%) of the 641 common mapped markers. The configuration type “D2.15”-“D2.15,” homozygous in the female parent and heterozygous for the male parent in both populations, was the second most frequent marker type, with 70 (10.92%) mapped markers. Finally, the configuration types with heterozygous parents in one of both populations were the less frequent, with 62 (9.67%) markers defined as “B3.7”-“D1.10,” 41 (6.39%) markers “D1.10”-“B3.7” and 19 (2.96%) described as “B3.7-B3.7.”

A high agreement was observed between LG assignment of the Discovery-identified SNPs, mapped in the composite map and the physical positions of these markers among the *E. grandis* chromosomes. This same result was observed in the single population maps. Out of 1,897 markers mapped in the composite map, 474 (24.98%) SNPs corresponded to markers identified by the Discovery pipeline, and of these, 437 (92.19%) showed correspondence between the assignment to a LGs in *A. sellowiana* and *E. grandis* chromosomes (Supplementary Data Sheet 4). Other 32 markers were mapped in discordant reference chromosomes and the remaining five were assigned to *E. grandis* genome scaffolds. Despite the high level of synteny observed, the marker order (colinearity) in the *A. sellowiana* composite map was not consistent with the physical positions in the *E. grandis* genome.

## 4. Discussion

Applied for the first time in *A. sellowiana*, the GBS approach demonstrated the advantage of coupled NGS with methods for reducing genome complexity to discover and genotype SNP markers. In our study, the rare-cutting enzyme *Pst*I was selected for targeting fewer sites with greater depth at each genomic site. As a result, although fewer SNPs were identified, a high site coverage was achieved, suitable for a full-sib mapping population of outcrossing species. It is relevant to note that this kind of populations present a high degree of linkage disequilibrium, so the number of markers required for covering the entire genome is relatively low. The total number of reads and average read per sample obtained in this study were comparable to the observed for other fruit tree species studies (Russell et al., 2014; Guajardo et al., 2015). However, these values are influenced not only by the restriction enzyme used but also by genome features of the species and the availability of a reference genome, among other factors (Gardner et al., 2014).

The original GBS protocol introduced the use of a sequenced reference genome to identify SNPs (Elshire et al., 2011). Considering that *A. sellowiana* lacks genome sequence information, the *E. grandis* genome was used as reference. As a result, an acceptable alignment value (30.1%) of *A. sellowiana* reads against the *E. grandis* genome was found. Using the same reference genome, the proportion of *E. urophylla* read alignment was twice larger (69.1%), as expected between more closely related species (Bartholomé et al., 2014). Differences in read alignment rates mainly depends on the quality of the genome assembly used as well as the phylogenetic relationship between the species. The occurrence of unaligned reads can be explained by poor-quality reads, poor-quality genome assembly, as well as the presence of divergent or species-specific sequences (Hyma et al., 2015). In this work, alignment distribution of *A. sellowiana* reads was homogeneous onto 11 *E. grandis* chromosomes (Figure 1). Chromosomes 8, 2, and 6 presented the highest number of identified SNPs, as was reported by Bartholomé et al. (2014), in the alignment of short reads obtained from the complete genome sequencing of *E. grandis* and *E. urophylla*. Although these chromosomes presented medium to large size, the number of identified markers had not a linear relationship with chromosome size. For instance, chromosome 5, which is one of the largest chromosome of *E. grandis*, presented a low *A. sellowiana* read alignment rates. This result can be explained by unique features of the *E. grandis* genome assembly (Bartholomé et al., 2014).

As a complementary strategy, the *de novo* based approach implemented in the UNEAK pipeline (Lu et al., 2013) was applied in this work. Using this approach, we identified ~3-fold fewer markers than the number of markers identified using the *E. grandis* as reference genome. This notable difference was also observed when comparing the mostly widespread applied reference-free with reference-based GBS pipelines (Torkamaneh et al., 2016). The low UNEAK-identified SNP number may result of a rather stringent network filter, which allowed only one mismatch for pair-read, may not only reduce the SNP number but also errors in genotype calling, caused by paralogous or repetitive sequences. The two strategies implemented in our study allowed to successfully identify a high number of SNP markers for *A. sellowiana* and represented a robust strategy for other non-reference species.

The correct genotype calling from the NGS data presents unique challenges for highly heterozygous species. The low read coverage of GBS data results in a large proportion of missing data as well as heterozygous undercalling. The latter may be due to unequal allele sampling or to a high sequencing base error rate of NGS reads (Swarts et al., 2014). Both, missing values and heterozygous undercalling, hinder linkage analysis, with a substantial impact in marker ordering and phasing, increasing the total map length. To overcome this limitation, new approaches have been developed to improve genotype calling in outcrossing species as well as to impute missing genotypes (Swarts et al., 2014; Covarrubias-Pazaran et al., 2016; Kim et al., 2016; Gerard et al., 2018). Here, we used a genotype calling method that takes advantages of the relative abundance of each allele (read counts) and the Mendelian properties of the mapping populations (Serang et al., 2012). To call genotypes with a high confidence, this methodology simultaneously considers all the genetic information available, as the parental information, the site coverage of each allele and the expected frequencies of individual genotype for each locus (Garcia et al., 2013). Despite the fact that it was initially designed for genotype calling in polyploid species, this quantitative genotyping analysis has proven to be an efficient approach to overcome the main constraints presented by genotyping in highly heterozygous species.

As a result of both SNP and genotyping calling approaches, a large number of high reliable SNP markers, useful for mapping purposes was obtained. However, the final number of useful SNPs retained represented a small proportion of those initially identified. Of the 55,000 SNP discovered, only 9.42% (5350) and 7.44% (4227) of them from H5 and H6 populations, respectively, were retained. This dramatic reduction has also been reported for other fruit tree species, where although many SNPs were identified, robust genotype calling were generated for only a small proportion of them (Gardner et al., 2014). Despite this reduction, our results are in concordance with the number of GBS-SNPs expected in full-sib mapping populations, and are superior to those found in apple and grape, where only 6.0 and 4.2% of the markers were retained for linkage mapping purposes (Gardner et al., 2014; Hyma et al., 2015). Both, Discovery and UNEAK approaches, equally contributed to the final SNP set. However, these approaches significantly differed at the initial number of markers as well as the proportion of retained markers across the filtering process. Thus, only 6.63 and 4.72% of the markers identified in the Discovery approach were selected for H5 and H6 populations, respectively. This proportion increased to 18.43% for H5 and 16.24% for H6 populations, for the UNEAK approach. This result shows that combining both SNP calling approaches with a quantitative genotyping method allowed to obtain high-quality markers covering the entire genome.

The first saturated genetic maps with 1,256 and 1,302 markers for H5 and H6 populations, respectively, were obtained for *A. sellowiana*. Both maps established 11 LGs, matching the expected haploid chromosome number of the species (*n* = 11). The total map length and average distance between markers was similar between maps, as was the number of mapped markers. In addition, the number of markers on both maps was comparable to the high-density maps published for other non-model tree fruit species (Ward et al., 2013; Russell et al., 2014; Guajardo et al., 2015). Nevertheless, the percentage of SNPs mapped from the initial data set was low (23.48% for H5 population and 30.80% for H6 population). This result could be explained by an uneven distribution of GBS-SNPs in the genome, resulting in large under-represented areas that could not be successfully covered in the genetic maps. Differences in the marker distribution could also explain the low number (23) of markers from the former H5 genetic map (Quezada et al., 2014) that could be mapped.

The comparison between single individual maps pointed out consistent pattern related to the total length and number of mapped markers among the LGs. For instance, both H5 and H6 LGs presented a slight variability in the number of mapped markers per LG, with the exception of the LG5 that presented a significantly lower number of markers. This result, also reported for other fruit tree species could be explained by a non-uniform distribution of SNP markers among chromosomes, occurrence of structural variations, as well by a local decrease of polymorphism in the same regions of the genome (Ward et al., 2013; Russell et al., 2014; Guajardo et al., 2015). Low proportion of markers with segregation distortion were observed in H5 (3.80%) and H6 (3.69%) maps, although distribution pattern for these markers was inconsistent between maps. Several reports have evaluated the impact of distorted segregation ratios in marker data on the construction of genetic linkage maps (Hackett and Broadfoot, 2003; Bodénès et al., 2016). However, the low number of distorted markers finally mapped is expected to have a minor impact in genetic distances, map length or marker order estimations, and therefore may not affect the construction of H5 or H6 genetic linkage maps.

The large number of common markers between H5 and H6 individual maps (51.86 and 49.23% of mapped markers in H5 and H6 map, respectively) was expected considering that both populations shared the same female parent. All the common markers had a complete correspondence with homologous LGs and showed a strong colinearity, confirming the robustness of the individual population maps. As commonly reported in mapping studies, some local inconsistencies in marker order were observed between these maps. The inconsistencies can be attributed to biological factors (such as chromosomal rearrangements, segregation distortion, and so on), sampling bias as well as to technical errors. Thus, structural variations have been reported in apple, where segmental duplications among different populations affect recombination frequencies, influencing marker order accuracy (Khan et al., 2012). In other study, local inconsistencies in marker order were attributed to a sampling bias for small mapping populations (*N* = 50) (Doligez et al., 2006). In our study, based on a comparative large mapping population (*N* = 160 and *N* = 184), the overall agreement between H5 and H6 marker order suggests that technical errors from genotyping methods and/or marker information content as the main causes of the local inconsistencies. The small inconsistencies observed can be a result of missing data, genotyping errors, or difference in content information among the markers and populations. Differences in recombination rates between the three parental genotypes were not considered, since they had little effect in incongruence of marker orders. Likewise, the effect of segregation distortion that can be considered another potential source of error in the ordering process was discarded because of the small proportion of distorted markers mapped.

Several mapping procedures for constructing composite genetic maps have been reported and applied to outcrossing species (Van Ooijen, 2006; Ronin et al., 2012; Endelman and Plomion, 2014). Despite differences in the statistical approaches, all of them are based on the combination of genetic distances estimation from single population maps. These approaches are negatively affected by differences in population sizes, marker information content, missing data, as well as the proportion of common markers. Consequently, marker order inconsistencies and map-distance inflation reduced the resolution and accuracy of the maps generated through these approaches (Doligez et al., 2006; De Keyser et al., 2010; Clark et al., 2014).

The innovative strategy to construct a composite map proposed in this work integrates the recombination information from the individuals of two genetically connected populations. The main advantage of our procedure is that the meiosis information of all individuals (*N* = 344) is captured in a single composite map, since the information data of common mapped markers was merged. Considering the complete correspondence between H5 and H6 LGs, the integration process was carried out for each LG independently. In a first step, only common markers were considered, with the objective of reducing missing information that hinders the marker ordering process (Hackett and Broadfoot, 2003). The resulting framework map provided the most accurate marker order that allowed the correction of the small order inconsistencies in the single population maps, resulting in robust high-density linkage maps. The improved estimation of marker positions can be explained by the larger number of recombination events evaluated in a composite approach, compared to single population approaches. Another possible explanation is that the multipoint maximum likelihood estimation was implemented using genetic information derived from two populations simultaneously, an efficient approach both for statistical and biological reasons. Although the two-point recombination fractions between common markers for H5 and H6 maps were estimated, the multipoint approach was preferred due to the higher accuracy of recombination fraction estimation (Mollinari et al., 2009). Finally, the incorporation of single population markers into the framework map enabled to saturate the composite map.

Using this strategy, a total of 1,897 markers were mapped in the high-density composite map, where 641 framework markers were common between H5 and H6 individual maps. This is by far the most saturated linkage map of *A. sellowiana* available to date. The composite genetic map resulted in a more comprehensive representation of the genome, including information of two mapping populations. This map included more markers than individuals ones, with a small total length (1,314 cM), reducing the average distance between markers from around 1.25 cM in the individual maps to 0.69 cM in the composite map. In addition, the composite map allowed the reduction of the number of gaps, to only three gaps larger than 15 cM. These results were in accordance with previous reports about the influence of population size in linkage maps, specifically in outcrossing species. For instance, lower marker densities were observed when small population size were analyzed (Bartholomé et al., 2014). Besides, high correlations were reported between population size and number of mapped markers (Hyma et al., 2015). The progeny size has a direct effect on the number of detected recombinants events, since smaller populations have fewer recombinants than larger populations. For H5 (*N* = 160) and H6 (*N* = 184) genetic maps, the mapping resolution achieved was in accordance with the mapping population sizes. The construction of the composite map is adequate to capture the genetic information of a larger population, generating a map with higher precision in the order and distance between markers. Besides, the segregation analysis using connected mapping populations might increase the coverage of the genome, filling the gaps and increasing the mapping resolution. Our results are consistent with previous reports that showed that the number of recombination events evaluated, thus the population size is the current limiting factor to construct high-density linkage maps in outcrossing species (Bartholomé et al., 2014; Hyma et al., 2015).

The level of synteny between *A. sellowiana* (*n* = 11) and *E. grandis* (*n* = 11) was examined through the distribution of Discovery-identified SNP markers, which were assigned to a chromosomal position in the *E. grandis* genome. A high degree of synteny was found between the two species, considering that more than 90% of the Discovery-identified markers were syntenic to *E. grandis* chromosomes. Although this result suggested the conserved localization of markers, colinearity (order of the markers) within each LG was not observed (Supplementary Data Sheet 4). It is important to highlight our results considering the phylogenetic divergence and the genome size difference between *A. sellowiana* (245 Mb) (da Costa et al., 2008) and *E. grandis* (641 Mb). However, these results are consistent with the high level of synteny observed within the Myrtaceae family (Grattapaglia et al., 2012). In addition, the recent comparison of chloroplast genomes of myrtle species, including *A. sellowiana* and *E. grandis* also revealed a highly conserved genome content, gene order and genomic structure between these species (de Machado et al., 2017). The high synteny observed in this preliminary study provides the basis for using the *E. grandis* reference genome in genomic studies of minor crops in the Myrtaceae family.

Here, the composite genetic map provided a reference for *A. sellowiana*, a valuable tool for future genetic and genomic applications. The composite map especially improved the accuracy of order marker and genetic distances, facilitating the comparison between genetic maps. This map will be also a useful tool to guide the assembly of the *A. sellowiana* genome sequence, as a reference to anchor and orient the sequence scaffolds. With the development of composite genetic maps, the occurrence of genome structural variation or conserved synteny can be evaluated across divergent species. To date, the comparative mapping studies in the Myrtaceae family had only included dry fruit species of the tribe Eucalypteae (Grattapaglia et al., 2012). Therefore, the composite map developed in this work can be useful to extend the studies including more divergent species of the family. In addition, this composite map may allow to align the position of QTLs detected across variable genetic backgrounds, facilitating the transfer of genetic information from molecular markers and gene positions, and accelerating molecular breeding strategies such as marker-assisted selection (Diaz et al., 2011).

## 5. Conclusions

In this study, we constructed the first high-density genetic map for *A. sellowiana* using a genotyping by sequencing approach. The GBS protocol was an effective strategy for simultaneous SNP discovery and genotyping, identifying thousands of genome-wide polymorphic markers, in a species with limited genetic resources. We also developed a novel strategy for constructing a composite genetic map using the genetic information from two full-sib connected mapping populations. This approach provided a better estimation of recombination fraction, resulted in higher accuracy for marker distance and order as well as increased genome coverage. The composite map along with the H5 and H6 single population maps are the most comprehensive representations of the *A. sellowiana* genome and constitute key genetic resources for this minor species. These maps could be useful for future genetic studies, such as the detection of QTL of important agronomic traits, comparative genome analysis in the Myrtaceae family, genome assembly, and the acceleration of the breeding process in *A. sellowiana*.

## Data Availability Statement

The original contributions presented in the study are publicly available. This data can be found here: https://www.ncbi.nlm.nih.gov/bioproject/PRJNA517479.

## Author Contributions

CP and AG conceived the study design. BV and DC provide the plant material. MQ performed the DNA extraction. MQ and AG designed the GBS experiments. MQ performed the GBS data analysis and the genetic map analysis. RA and MQ carried out the novel genetic map analysis, wrote computer code, and integrated the analysis of results. MQ wrote the manuscript draft. CP, RA, and AG edited and revised the manuscript. All authors read and approved the final manuscript.

## Conflict of Interest

The authors declare that the research was conducted in the absence of any commercial or financial relationships that could be construed as a potential conflict of interest.
