# Supplementary material for: Construction of a High-Density Genetic Map of Acca sellowiana (Berg.) Burret, an Outcrossing Species, Based on Two Connected Mapping Populations
Source: Front Plant Sci. 2021 Feb 23;12:626811. doi: 10.3389/fpls.2021.626811 (PMC7940835; doi:10.3389/fpls.2021.626811)
Supplement: Supplementary file 1 [file Data_Sheet_1.pdf]

frontiers

Supplementary Material

Supplementary Material of **"Construction of a high-density genetic map of *Acca sellowiana* (Berg.) Burret, an outcrossing species, based on two connected mapping populations".**

Here, we illustrate how to obtain maximun likelihood estimates of two-point and multipoint recombination fractions.

1 TWO POINTS RECOMBINATION FRACTION

The populations are composed by two full-sibling populations with a common mother: one full-sibling population derived from the cross of ♀ × ♂^{γ} and one full-sibling population derived from the cross of ♀ × ♂^{β}.

1.1 Informative mother and non-informative fathers

The first example is about how to estimate the two point recombination fraction in a case where the ♀ loci are informative and the ♂ loci is not. Following Wu et al. (2002) notation, a marker D1.10 is a marker with the cross type ab × aa, *i.e.*, the cross between one heterozygous (ab) and homozygous (aa) genotypes. Assuming the first parent as the mother, the configuration is D1.10-D1.10. Here we assume a D1.10 situation in both populations (♀ × ♂^{γ} and ♀ × ♂^{β}) for the same two loci. The superscript represents the father and the subscript the loci position. The superscript is supressed for the maternal alleles.

The cross is then

$$\begin{array}{ccccc} \text{♂}^{\gamma} & & \text{♀} & & \text{♂}^{\beta} \\ \left.\begin{array}{c} a_1^{\gamma} \\ \\ a_2^{\gamma} \end{array}\right\| \left|\begin{array}{c} a_1^{\gamma} \\ \\ a_2^{\gamma} \end{array}\right. & \times & \left.\begin{array}{c} a_1 \\ \\ a_2 \end{array}\right\| \left|\begin{array}{c} b_1 \\ \\ b_2 \end{array}\right. & \times & \left.\begin{array}{c} a_1^{\beta} \\ \\ a_2^{\beta} \end{array}\right\| \left|\begin{array}{c} a_1^{\beta} \\ \\ a_2^{\beta} \end{array}\right. \end{array}$$

Gametes after meiosis for each parent:

$$\begin{array}{ccccc} \text{♂}^{\gamma} & & \text{♀} & & \text{♂}^{\beta} \\ \left.\begin{array}{c} a_1^{\gamma} \\ \\ a_2^{\gamma} \end{array}\right| & \times & \left.\begin{array}{c} a_1 \\ \\ a_2 \end{array}\right| \quad \left.\begin{array}{c} b_1 \\ \\ b_2 \end{array}\right| \quad \left.\begin{array}{c} a_1 \\ \\ b_2 \end{array}\right| \quad \left.\begin{array}{c} b_1 \\ \\ a_2 \end{array}\right| & \times & \left.\begin{array}{c} a_1^{\beta} \\ \\ a_2^{\beta} \end{array}\right| \end{array}$$

Recombination fractions of each gamete (in this case, just meiosis from the ♀ parent is informative):

$$\begin{array}{cccc} & \multicolumn{2}{c}{♀} & \\ \frac{1-r}{2} & \frac{1-r}{2} & \frac{r}{2} & \frac{r}{2} \end{array}$$

Counting recombinants:

$$\begin{array}{cccc} & \multicolumn{2}{c}{♀} & \\ n_1 & n_2 & n_3 & n_4 \end{array}$$

The likelihood function of the recombination fraction based on the cross $♂^{\gamma} \times ♀$:

$$L(r^{\gamma}) = \left(\frac{1-r}{2}\right)^{n_1^{\gamma}+n_2^{\gamma}} \left(\frac{r}{2}\right)^{n_3^{\gamma}+n_4^{\gamma}}$$

The maximum likelihood estimator (MLE) of the recombination fraction (r^{γ}) can be obtained by first applying a logarithm to $L(r^{\gamma})$:

$$\log L(r^{\gamma}) = l(r^{\gamma}) = (n_1^{\gamma} + n_2^{\gamma})\log\left(\frac{1-r}{2}\right) + (n_3^{\gamma} + n_4^{\gamma})\log\left(\frac{r}{2}\right)$$

The first derivative $l'(r^{\gamma})$:

$$l'(r^{\gamma}) = (n_1^{\gamma} + n_2^{\gamma})\left(\frac{2}{1-r}\right)(-1) + (n_3^{\gamma} + n_4^{\gamma})\left(\frac{2}{r}\right)$$

Making it equals to 0 and solving for r provides the MLE of r^{γ}:

$$\hat{r}^{\gamma} = \frac{n_3^{\gamma} + n_4^{\gamma}}{n_1^{\gamma} + n_2^{\gamma} + n_3^{\gamma} + n_4^{\gamma}}$$

It is easy to understand that, as expected, for this marker configuration, $\hat{r}^{\gamma}$ is obtained by a ratio of recombinants over the total number of individuals. For other configurations, one can simply change the numerator by the expected number of recombinations. This is the core of the EM algorithm for multipoint analysis, as will be shown.

Using the same reasoning, the likelihood function of the recombination fraction based on the cross $\male^{\beta} \times \female$:

$$L(r^{\beta}) = \left(\frac{1-r}{2}\right)^{n_1^{\beta}+n_2^{\beta}} \left(\frac{r}{2}\right)^{n_3^{\beta}+n_4^{\beta}}$$

The MLE:

$$\hat{r}^{\beta} = \frac{n_3^{\beta} + n_4^{\beta}}{n_1^{\beta} + n_2^{\beta} + n_3^{\beta} + n_4^{\beta}}$$

Assuming that the recombination fraction in one population is independent of the recombination in the other (since we are measuring recombination in the $\female$ parent in two independent experiments), the joint likelihood function will be:

$$L(r^{\gamma\beta}) = \left(\frac{1-r}{2}\right)^{n_1^{\gamma}+n_2^{\gamma}} \left(\frac{r}{2}\right)^{n_3^{\gamma}+n_4^{\gamma}} \times \left(\frac{1-r}{2}\right)^{n_1^{\beta}+n_2^{\beta}} \left(\frac{r}{2}\right)^{n_3^{\beta}+n_4^{\beta}}$$

Therefore,

$$L(r^{\gamma\beta}) = \left(\frac{1-r}{2}\right)^{n_1^{\gamma}+n_2^{\gamma}+n_1^{\beta}+n_2^{\beta}} \left(\frac{r}{2}\right)^{n_3^{\gamma}+n_4^{\gamma}+n_3^{\beta}+n_4^{\beta}}$$

And after applying log and taking derivatives:

$$\hat{r}^{\gamma\beta} = \frac{n_3^{\gamma} + n_4^{\gamma} + n_3^{\beta} + n_4^{\beta}}{n_1^{\gamma} + n_2^{\gamma} + n_3^{\gamma} + n_4^{\gamma} + n_1^{\beta} + n_2^{\beta} + n_3^{\beta} + n_4^{\beta}}$$

This shows that the combined two-point MLE is simply obtained by counting recombinants in both populations, over the total number of individuals.

2 MULTIPOINT RECOMBINATION FRACTION

Under the same assumptions, it is possible to derive an Expectation and Maximization (EM) algorithm to estimates the multipoint recombination fraction, expanding Wu et al. (2002) approach. All that follows is just a generalization of the MLE for $\hat{r}^{\gamma\beta}$.

In Wu et al. (2002) was proposed a general maximum-likelihood-based algorithm for simultaneously estimating linkage and linkage phases in outcrossing species. The maximum likelihood estimator of the recombination fraction for two markers is estimated in an iterative procedure based on Expectation and Maximization (EM) algorithms Lander and Green (1987); Dempster et al. (1977). For outcrossing populations, the linkage phase between markers is commonly unknown and needs to be estimated. This approach is implemented in ONEMAP R package Margarido et al. (2007).

In this study, the EM equations presented by Wu et al. (2002) were adapted to estimate the recombination fractions of two connected populations. Our model will be described using the same notation, as well as the matrix **H**, **D** and **I**. The possible assignment of linkage phases between the two parental genotypes for two adjacent markers were denoted as A^{ω}, where $\omega = 1, \ldots, 4$ (coupling $\times$ coupling, coupling $\times$ repulsion, repulsion $\times$ coupling, repulsion $\times$ repulsion, respectively).

The statistical model presented here assumed two connected F_1 mapping populations (denoted as N and N' of sample size j and j', respectively) genotyped with the same marker set. First, the recombination fraction was estimated in each single-population using the approach of Wu et al. (2002). This was used as the initial value for the joint recombination fraction estimation using EM algorithm. The adapted equations are given below.

E step: The expectation step is independently computed on each population as follows:

- N population: at step τ, the matrix $\mathbf{H}^{\omega(\tau)}_{k(k+1)}$ based on the current recombination fraction ($\vartheta^{\omega(\tau)}_{k(k+1)}$) is used to calculate the expected number of recombination events between adjacent marker loci M_k and M_{k+1} for offspring j under the phase assignment $\mathsf{A}^{\omega}_{k+1}$,

$$c^{\omega\{\tau+1\}}_{i_k i_{k+1} j} = \frac{\mathbf{m}^{\mathrm{T}}_{i_k j}[\mathbf{I}^{\mathrm{T}}_{p_k}(\mathbf{D}^{\omega}_{k(k+1)} \circ \mathbf{H}^{\omega\{\tau\}}_{k(k+1)})\mathbf{I}_{p_{k+1}}]\mathbf{m}_{i_{k+1} j}}{\mathbf{m}^{\mathrm{T}}_{i_k j}\mathbf{P}^{\omega\{\tau\}}_{k(k+1)}\mathbf{m}_{i_{k+1} j}}$$

where

$\mathbf{m}_{i_k j}$, is the p_k-dimensional vector of the indicator variable for marker M_k
$\mathbf{I}_{p_k}$, is a $(4 \times p_k)$ incidence matrix relating the parental chromosome pairings genotypes to phenotypes
$\mathbf{D}^{\omega}_{k(k+1)}$, is a (4×4) matrix of the number of recombination events between markers M_k and M_{k+1}
$\mathbf{H}^{\omega}_{k(k+1)}$, is the (4×4) matrix of the transition probability of the parental chromosome pairings from markers M_k to M_{k+1}
$\mathbf{P}^{\omega}_{k(k+1)} = \mathbf{I}^{\mathrm{T}}_{p_k}\mathbf{H}^{\omega}_{k(k+1)}\mathbf{I}_{p_{k+1}}$, is a $(p_k \times p_{k+1})$ matrix of the transition probability from markers M_k to M_{k+1}

- N' population: at step τ, the matrix $\hat{\mathbf{H}}^{\hat{\omega}(\tau)}_{k(k+1)}$ based on the current recombination fraction ($\hat{\vartheta}^{\hat{\omega}(\tau)}_{k(k+1)}$) was used to calculate the expected number of recombination events between M_k and M_{k+1} for offspring j' under assignment $\mathsf{A}^{\hat{\omega}}_{k+1}$,

$$\hat{c}^{\hat{\omega}\{\tau+1\}}_{i_k i_{k+1} j'} = \frac{\hat{\mathbf{m}}^{\mathrm{T}}_{i_k j'}[\hat{\mathbf{I}}^{\mathrm{T}}_{\hat{p}_k}(\hat{\mathbf{D}}^{\hat{\omega}}_{k(k+1)} \circ \hat{\mathbf{H}}^{\hat{\omega}\{\tau\}}_{k(k+1)})\hat{\mathbf{I}}_{\hat{p}_{k+1}}]\hat{\mathbf{m}}_{i_{k+1} j'}}{\hat{\mathbf{m}}^{\mathrm{T}}_{i_k j'}\hat{\mathbf{P}}^{\hat{\omega}\{\tau\}}_{k(k+1)}\hat{\mathbf{m}}_{i_{k+1} j'}}$$

where

$\hat{\mathbf{m}}_{i_k j'}$, is the $\hat{p}_k$-dimensional vector of the indicator variable for marker M_k
$\hat{\mathbf{I}}_{\hat{p}_k}$, is a $(4 \times \hat{p}_k)$ incidence matrix relating the parental chromosome pairings genotypes to phenotypes
$\hat{\mathbf{D}}^{\hat{\omega}}_{k(k+1)}$, is a (4×4) matrix of the number of recombination events between the two markers M_k

and M_{k+1}
$\hat{\mathbf{H}}^{\hat{\omega}}_{k(k+1)}$, is the (4×4) matrix of the transition probability of the parental chromosome pairings from markers M_k to M_{k+1}
$\hat{\mathbf{P}}^{\hat{\omega}}_{k(k+1)} = \hat{\mathbf{I}}^{\mathrm{T}}_{\hat{p}_k} \hat{\mathbf{H}}^{\hat{\omega}}_{k(k+1)} \hat{\mathbf{I}}_{\hat{p}_{k+1}}$, is a $(\hat{p}_k \times \hat{p}_{k+1})$ matrix of the transition probability from markers M_k to M_{k+1}

M step: The maximization step is computed jointly for both populations. A single recombination value is estimated using the information of both populations simultaneously as follows:

$$\vartheta^{\omega\{\tau+1\}}_{k(k+1)} = \hat{\vartheta}^{\hat{\omega}\{\tau+1\}}_{k(k+1)} = \frac{1}{2N} \sum_{j=1}^{N} \sum_{i_k=1}^{p_k} \sum_{i_{k+1}=1}^{p_{k+1}} c^{\omega\{\tau+1\}}_{i_k i_{k+1} j} + \frac{1}{2N'} \sum_{j'=1}^{N'} \sum_{i_k=1}^{\hat{p}_k} \sum_{i_{k+1}=1}^{\hat{p}_{k+1}} \hat{c}^{\hat{\omega}\{\tau+1\}}_{i_k i_{k+1} j'}$$

The iterative procedure is repeated between the E and M step until the recombination fraction value converges to a stable value. Notice that ϑ is just a generalization of $\hat{r}^{\gamma\beta}$, considering markers with differente amount of information; also, it is a multipoint estimator.

Likelihood: Based on the independence between the maps, the likelihood of the composite map can be given by the product of the likelihood of the maps from the individuals populations as given in Wu et al. (2002):

$$P(\mathbf{M}|\mathbf{A}_{\omega_2\omega_3\ldots\omega_4}, \mathbf{A}_{\hat{\omega}_2\hat{\omega}_3\ldots\hat{\omega}_4}) = \prod_{j=1}^{N} \prod_{k=2}^{m} [\mathbf{m}^T_{i_{k-1}j} \mathbf{P}^{(k-1)k}_{\omega_k} \mathbf{m}_{i_k j}] \prod_{j'=1}^{N'} \prod_{k=2}^{m} [\hat{\mathbf{m}}^T_{i_{k-1}j'} \hat{\mathbf{P}}^{(k-1)k}_{\hat{\omega}_k} \hat{\mathbf{m}}_{i_k j'}]$$

REFERENCES

Dempster, A., Laird, N., and Rubin, D. B. (1977). Maximum likelihood from incomplete data via the EM algorithm. *J. R. Stat. Soc, Series B: Methodol.* 39, 1–22. doi:10.1111/j.2517-6161.1977.tb01600.x

Lander, E. S. and Green, P. (1987). Construction of multilocus genetic linkage maps in humans. *Proc. Natl. Acad. Sci.* 84, 2363–2367. doi:10.1073/pnas.84.8.2363

Margarido, G. R. A., de Souza, A. P., and Garcia, A. A. F. (2007). Onemap: software for genetic mapping in outcrossing species. *Hereditas* 144, 78–79. doi:10.1111/j.2007.0018-0661.02000.x

Wu, R., Ma, C.-X., Painter, I., and Zeng, Z.-B. (2002). Simultaneous maximum likelihood estimation of linkage and linkage phases in outcrossing species. *Theor. Popul. Biol.* 61, 349–363. doi:10.1006/tpbi.2002.1577
