# Supplementary material for: Construction of a High-Density Genetic Map of Acca sellowiana (Berg.) Burret, an Outcrossing Species, Based on Two Connected Mapping Populations
Source: Front Plant Sci. 2021 Feb 23;12:626811. doi: 10.3389/fpls.2021.626811 (PMC7940835; doi:10.3389/fpls.2021.626811)
Supplement: Supplementary file 2 [file Data_Sheet_2.pdf]

onemap2pop

Tutorial to estimate recombination fraction and best order for two connected populations

Overview

Here we show how to use the HMM-EM algorithm of Quezada et al. (2021) implemented in the `onemap` R package to estimate the recombination fraction in a scenario with two outcrossing connected populations (having a common parent). The objective is, based on the information of both populations, to obtain the most likely order and multipoint distances.

Citation

To cite this R tutorial:

Quezada et al. (2021). Construction of a high-density genetic map of *Acca sellowiana* (Berg.) Burret, an outcrossing species, based on two connected mapping populations. *Front. Plant. Sci.* doi: 10.3389/fpls.2021.626811

Before to follow this tutorial

We expected that you have enough knowledge to build a linkage map for outcrossing populations with `onemap` software. If not, please follow its tutorial, available at http://augusto-garcia.github.io/onemap/vignettes_highres/Outcrossing_Populations.html.

Built-in data

In this tutorial, we will use a built-in data of the `onemap` package called `onemap2pop`. It is a simulated data of two full-sib populations that share one same parent. We used the software `PedigreeSim` (Voorrips and Maliepaard, 2012) to simulate them and `onemap` to build the individual linkage maps. To load this data:

```
data(onemap2pop)
```

rf_2pops

The function `rf_2pops` estimates the recombination fraction based on two mapping populations. It estimates the recombination fractions based on a multipoint approach implemented using the methodology of Hidden Markov Models (HMM) with the Expectation Maximization (EM) algorithm as explained in the supplementary material of Quezada et al. (2021).

To use it, the user must had already built the individual maps for each population and assigned the correspondent linkage groups within markers. After building the maps for each population, the user must present an initial order with sharable markers between both populations, i.e., both populations have the markers provided in this order. Let's assume that we built the following two linkage maps for a given linkage group (hereafter LG1) based on the information derived from two populations (`POP1` and `POP2`).

```
LG1_POP1_final
```

```
##
## Printing map:
##
```

```
## Markers           Position           Parent 1       Parent 2
##
##  1 M1                0.00           a |  | a       a |  | b
##  2 M2                5.35           a |  | a       a |  | b
##  3 M3                9.36           a |  | a       a |  | b
##  4 M4               20.97           a |  | b       a |  | b
##  5 M5               26.13           a |  | b       b |  | a
##  6 M6               30.29           a |  | b       a |  | b
##  7 M7               34.10           a |  | a       a |  | b
##  8 M8               37.44           a |  | a       a |  | b
##  9 M9               41.89           a |  | a       b |  | a
## 11 M11              49.11           a |  | a       a |  | b
## 12 M12              51.34           a |  | a       b |  | a
## 13 M13              57.46           a |  | a       a |  | b
## 10 M10              57.46           a |  | b       a |  | a
## 14 M14              69.49           a |  | b       b |  | a
## 15 M15              75.26           b |  | a       b |  | a
## 16 M16              80.24           a |  | b       a |  | a
## 17 M17              87.43           a |  | a       a |  | b
## 18 M18              90.47           a |  | a       b |  | a
## 19 M19              96.01           a |  | b       b |  | a
## 20 M20              99.88           b |  | a       a |  | a
## 21 M21             106.46           a |  | a       a |  | b
##
## 21 markers            log-likelihood: -1114.715
```

```
LG1_POP2_final
```

```
##
## Printing map:
##
## Markers           Position           Parent 1       Parent 2
##
##  1 M1                0.00           a |  | a       a |  | b
##  2 M2                6.71           a |  | a       a |  | b
##  3 M3                8.71           a |  | a       a |  | b
##  4 M4               13.41           a |  | b       a |  | b
##  5 M5               15.94           a |  | b       a |  | a
##  6 M6               24.65           a |  | b       b |  | a
##  7 M7               28.74           a |  | a       b |  | a
##  8 M8               30.34           a |  | a       a |  | b
##  9 M9               35.98           a |  | a       b |  | a
## 10 M10              44.36           a |  | b       a |  | b
## 11 M11              49.45           a |  | a       b |  | a
## 12 M12              56.79           a |  | a       b |  | a
## 13 M13              59.85           a |  | a       b |  | a
## 14 M14              62.74           a |  | b       a |  | b
## 15 M15              73.32           b |  | a       a |  | a
## 16 M16              76.78           a |  | b       a |  | a
## 17 M17              76.78           a |  | a       b |  | a
## 18 M18              81.91           a |  | a       a |  | b
## 20 M20              91.39           b |  | a       b |  | a
## 19 M19              95.08           a |  | b       a |  | a
## 21 M21              95.08           a |  | a       b |  | a
##
```

```
## 21 markers            log-likelihood: -1053.239
```

We have in this example two different orders for the same markers, one for each population:

```
LG1_POP1_final$seq.num
```

```
##  [1]  1  2  3  4  5  6  7  8  9 11 12 13 10 14 15 16 17 18 19 20 21
```

```
LG1_POP2_final$seq.num
```

```
##  [1]  1  2  3  4  5  6  7  8  9 10 11 12 13 14 15 16 17 18 20 19 21
```

The first step is to obtain the multipoint recombination fraction for the the two previously order based on the information of both populations.

```
## Extracting the marker names:
order_LG1POP1 <- colnames(POP1_geno$geno)[LG1_POP1_final$seq.num]

## Computing the rf and likelihood considering information of POP1 and POP2
LG1_POP1order <- rf_2pops(markers_names = order_LG1POP1,
                          data_P1 = POP1_geno,
                          data_P2 = POP2_geno,
                          rftwopoints_P1 = twopts_POP1,
                          rftwopoints_P2 = twopts_POP2,
                          LOD = 3,
                          max.rf = 0.5,
                          log10.mintol = -6,
                          max_it = 60)
```

```
## interaction: 10 ; loglike: -2261.52695815027 ; tol: 0.09

## interaction: 20 ; loglike: -2256.31269345973 ; tol: 0.01

## interaction: 30 ; loglike: -2251.8721010692 ; tol: 0.005

## interaction: 40 ; loglike: -2251.36224392631 ; tol: 7e-04

## interaction: 50 ; loglike: -2251.35050257312 ; tol: 6e-05

## interaction: 60 ; loglike: -2251.35040251465 ; tol: 5e-06
```

```
## Extracting the sequence likelihood of the order:
LG1_POP1order$P1P2_seq.like
```

```
## [1] -2251.35
```

```
## Extracting the marker names:
order_LG1POP2 <- colnames(POP2_geno$geno)[LG1_POP2_final$seq.num]

## Computing the rf and likelihood considering information of POP1 and POP2
LG1_POP2order <- rf_2pops(markers_names = order_LG1POP2,
                          data_P1 = POP1_geno,
                          data_P2 = POP2_geno,
                          rftwopoints_P1 = twopts_POP1,
                          rftwopoints_P2 = twopts_POP2,
                          LOD = 3,
                          max.rf = 0.5,
                          log10.mintol = -6,
                          max_it = 60)
```

```
## interaction: 10 ; loglike: -2178.64809140789 ; tol: 0.1
```

```
## interaction: 20 ; loglike: -2178.29040139245 ; tol: 0.02
## interaction: 30 ; loglike: -2171.05600028205 ; tol: 0.005
## interaction: 40 ; loglike: -2170.56243120964 ; tol: 7e-04
## interaction: 50 ; loglike: -2170.56057851398 ; tol: 6e-05
## interaction: 60 ; loglike: -2170.56057710544 ; tol: 5e-06
```

```
## Extracting the sequence likelihood of the order:
LG1_POP2order$P1P2_seq.like
```

```
## [1] -2170.561
```

The likelihood of the populations can not necessarily be comparable (due do differente sample sizes, missing data, informativeness of markers), but just to have a starting point, let us use the order of POP2 (higher likelihood) for both populations. To print the maps with such order:

```
LG1_POP2order
```

```
## $P1
##
## Printing map:
##
## Markers           Position           Parent 1       Parent 2
##
##  1 M1                 0.00           a |  | a       a |  | b
##  2 M2                 6.71           a |  | a       a |  | b
##  3 M3                 8.71           a |  | a       a |  | b
##  4 M4                13.41           a |  | b       a |  | b
##  5 M5                15.94           a |  | b       b |  | a
##  6 M6                24.65           a |  | b       a |  | b
##  7 M7                28.75           a |  | a       a |  | b
##  8 M8                30.34           a |  | a       a |  | b
##  9 M9                35.98           a |  | a       b |  | a
## 10 M10               44.35           a |  | b       a |  | a
## 11 M11               49.46           a |  | a       a |  | b
## 12 M12               56.82           a |  | a       b |  | a
## 13 M13               59.88           a |  | a       a |  | b
## 14 M14               62.73           a |  | b       b |  | a
## 15 M15               71.93           b |  | a       b |  | a
## 16 M16               74.99           a |  | b       a |  | a
## 17 M17               79.90           a |  | a       a |  | b
## 18 M18               84.30           a |  | a       b |  | a
## 20 M20               92.23           b |  | a       a |  | a
## 19 M19               94.70           a |  | b       b |  | a
## 21 M21               97.27           a |  | a       a |  | b
##
## 21 markers            log-likelihood: -1118.765
##
##
## $P2
##
## Printing map:
##
## Markers           Position           Parent 1       Parent 2
##
```

```
##  1 M1                 0.00          a |   | a       a |   | b
##  2 M2                 6.71          a |   | a       a |   | b
##  3 M3                 8.71          a |   | a       a |   | b
##  4 M4                13.41          a |   | b       a |   | b
##  5 M5                15.94          a |   | b       a |   | a
##  6 M6                24.65          a |   | b       b |   | a
##  7 M7                28.75          a |   | a       b |   | a
##  8 M8                30.34          a |   | a       a |   | b
##  9 M9                35.98          a |   | a       b |   | a
## 10 M10               44.35          a |   | b       a |   | b
## 11 M11               49.46          a |   | a       b |   | a
## 12 M12               56.82          a |   | a       b |   | a
## 13 M13               59.88          a |   | a       b |   | a
## 14 M14               62.73          a |   | b       a |   | b
## 15 M15               71.93          b |   | a       a |   | a
## 16 M16               74.99          a |   | b       a |   | a
## 17 M17               79.90          a |   | a       b |   | a
## 18 M18               84.30          a |   | a       a |   | b
## 20 M20               92.23          b |   | a       b |   | a
## 19 M19               94.70          a |   | b       a |   | a
## 21 M21               97.27          a |   | a       b |   | a
##
## 21 markers            log-likelihood: -1051.795
##
##
## $P1P2_seq.like
## [1] -2170.561
```

The Parent 1 is the common parent between the populations, therefore, has the same linakge phase configuration. Parent 2 is different between the populations, and so is free phase configuration. The recombination fraction on the maps is the one estimated using the information of both populations based on HMM-EM from Quezada et al. (2021). The log-likelihood is computed for each map using the same recombination fractions for POP1, POP2, and POP1 and POP2 simultaneously.

We will use the RIPPLE algorithm. This function is current not optimized and may take an overnight for each linkage group. To avoid such waiting in this tutorial, the object `ripple_result_LG1` was already made available and the user does not need to run the following chunk.

```
## It may take an overnight to run...
ripple_result_LG1 <- ripple_2pops(markers_names = order_LG1POP2,
                                  data_P1 = POP1_geno,
                                  data_P2 = POP2_geno,
                                  twopts_POP1 = twopts_POP1,
                                  twopts_POP2 = twopts_POP2,
                                  LOD = 3,
                                  max.rf = 0.5,
                                  log10.mintol = -2,
                                  max_it = 60,
                                  window = 4)
```

Now we find the order that maximizes the log-likelihood of the map.

```
## Which rippled order has the higher likelihood
max(ripple_result_LG1[[2]])
```

```
## [1] -2169.025
```

```
## Which is such order
which(ripple_result_LG1[[2]]==max(ripple_result_LG1[[2]]))[1]
```

```
## [1] 386
```

```
## Creating an object with such order
final_order_LG1 <- ripple_result_LG1[[1]][386,]
```

Based on the RIPPLE results, the 386 has the highest likelihood which is also higher than the initial order from the POP2 map. Therefore, we will use it as our final linkage group order. It is worthy noting that this order matches with the one we simulated. Building and printing our final order of LG1:

```
LG1_final <- rf_2pops(markers_names = final_order_LG1,
                      data_P1 = POP1_geno,
                      data_P2 = POP2_geno,
                      rftwopoints_P1 = twopts_POP1,
                      rftwopoints_P2 = twopts_POP2,
                      LOD = 3,
                      max.rf = 0.5,
                      log10.mintol = -6,
                      max_it = 60)
```

```
LG1_final
```

```
## $P1
##
## Printing map:
##
## Markers            Position           Parent 1       Parent 2
##
##   1 M1                 0.00           a |  | a       a |  | b
##   2 M2                 6.71           a |  | a       a |  | b
##   3 M3                 8.71           a |  | a       a |  | b
##   4 M4                13.41           a |  | b       a |  | b
##   5 M5                15.94           a |  | b       b |  | a
##   6 M6                24.65           a |  | b       a |  | b
##   7 M7                28.75           a |  | a       a |  | b
##   8 M8                30.34           a |  | a       a |  | b
##   9 M9                35.98           a |  | a       b |  | a
##  10 M10               44.34           a |  | b       a |  | a
##  11 M11               49.46           a |  | a       a |  | b
##  12 M12               56.82           a |  | a       b |  | a
##  13 M13               59.89           a |  | a       a |  | b
##  14 M14               62.73           a |  | b       b |  | a
##  15 M15               71.58           b |  | a       b |  | a
##  16 M16               74.55           a |  | b       a |  | a
##  17 M17               81.04           a |  | a       a |  | b
##  18 M18               85.64           a |  | a       b |  | a
##  19 M19               91.56           a |  | b       b |  | a
##  20 M20               93.85           b |  | a       a |  | a
##  21 M21               98.76           a |  | a       a |  | b
##
## 21 markers            log-likelihood: -1110.359
##
##
## $P2
```

```
##
## Printing map:
##
## Markers            Position           Parent 1       Parent 2
##
##  1 M1                  0.00           a |  | a       a |  | b
##  2 M2                  6.71           a |  | a       a |  | b
##  3 M3                  8.71           a |  | a       a |  | b
##  4 M4                 13.41           a |  | b       a |  | b
##  5 M5                 15.94           a |  | b       a |  | a
##  6 M6                 24.65           a |  | b       b |  | a
##  7 M7                 28.75           a |  | a       b |  | a
##  8 M8                 30.34           a |  | a       a |  | b
##  9 M9                 35.98           a |  | a       b |  | a
## 10 M10                44.34           a |  | b       a |  | b
## 11 M11                49.46           a |  | a       b |  | a
## 12 M12                56.82           a |  | a       b |  | a
## 13 M13                59.89           a |  | a       b |  | a
## 14 M14                62.73           a |  | b       a |  | b
## 15 M15                71.58           b |  | a       a |  | a
## 16 M16                74.55           a |  | b       a |  | a
## 17 M17                81.04           a |  | a       b |  | a
## 18 M18                85.64           a |  | a       a |  | b
## 19 M19                91.56           a |  | b       a |  | a
## 20 M20                93.85           b |  | a       b |  | a
## 21 M21                98.76           a |  | a       b |  | a
##
## 21 markers            log-likelihood: -1052.619
##
##
## $P1P2_seq.like
## [1] -2162.978
```

This procedure needs to be applied for all the other linkage groups.

References

Voorrips, R. E., Maliepaard, C. A. (2012). The simulation of meiosis in diploid and tetraploid organisms using various genetic models. *BMC Bioinformatics* 13, 248. doi:10.1186/1471-2105-13-248

Quezada, M., Amadeu, R.R., Vignale, B., Cabrera, D., Pritsch, C., Garcia, A.A.F. (2021). Construction of a high-density genetic map of *Acca sellowiana* (Berg.) Burret, an outcrossing species, based on two connected mapping populations. *Front. Plant. Sci.* doi: 10.3389/fpls.2021.626811
