# Supplementary material for: Construction of a High-Density Genetic Map of Acca sellowiana (Berg.) Burret, an Outcrossing Species, Based on Two Connected Mapping Populations
Source: Front Plant Sci. 2021 Feb 23;12:626811. doi: 10.3389/fpls.2021.626811 (PMC7940835; doi:10.3389/fpls.2021.626811)
Supplement: Supplementary file 3 [file Data_Sheet_3.pdf]

Supplementary Material

Supplementary Material of **“Construction of a high-density genetic map of *Acca sellowiana* (Berg.) Burret, an outcrossing species, based on two connected mapping populations”.**

SUPPLEMENTARY FILE 3

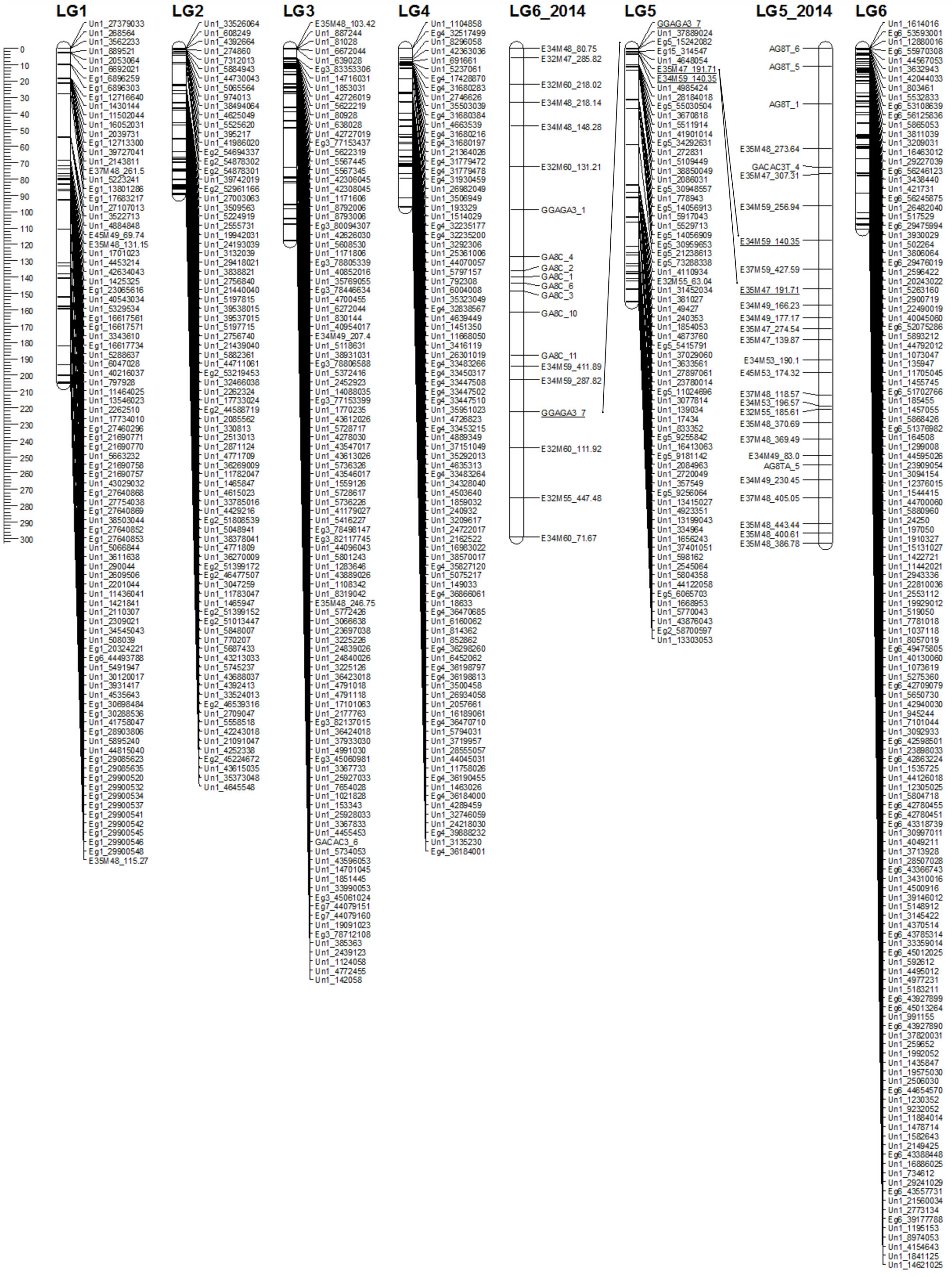

Figure S3. Comparison of homologous linkage groups from H5 genetic maps. Linkage groups derived from the current work were aligned with the corresponding linkage groups developed by (Quezada et al., 2014) (identified as 2014). Common markers between groups are underlined and connected with a line. Map distances (cM) are indicated at the left side of the figure

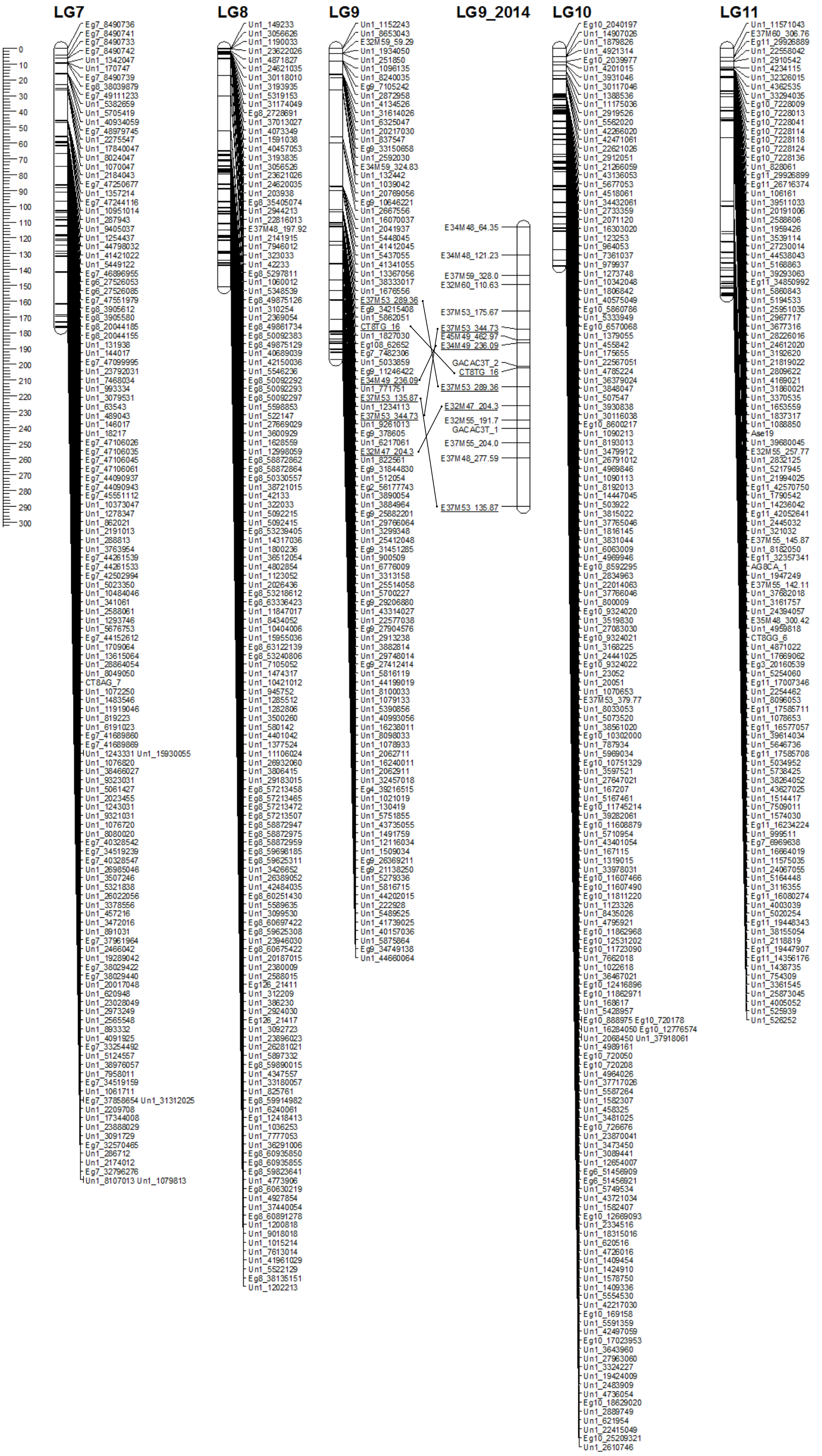

Figure S3 (Cont.). Comparison of homologous linkage groups from H5 genetic maps. Linkage groups derived from the current work were aligned with the corresponding linkage groups developed by (Quezada et al., 2014) (identified as 2014). Common markers between groups are underlined and connected with a line. Map distances (cM) are indicated at the left side of the figure.

REFERENCES

Quezada, M., Pastina, M. M., Ravest, G., Silva, P., Vignale, B., Cabrera, D., et al. (2014). A first genetic map of *Acca sellowiana* based on ISSR, AFLP and SSR markers. *Sci. Hortic.* 169, 138–146. doi:doi.org/10.1016/j.scienta.2014.02.009
