# Supplementary material for: Construction of a High-Density Genetic Map of Acca sellowiana (Berg.) Burret, an Outcrossing Species, Based on Two Connected Mapping Populations
Source: Front Plant Sci. 2021 Feb 23;12:626811. doi: 10.3389/fpls.2021.626811 (PMC7940835; doi:10.3389/fpls.2021.626811)
Supplement: Supplementary file 4 [file Data_Sheet_4.pdf]

Supplementary Material

Supplementary Material of **"Construction of a high-density genetic map of *Acca sellowiana* (Berg.) Burret, an outcrossing species, based on two connected mapping populations"**.

SUPPLEMENTARY FILE 4

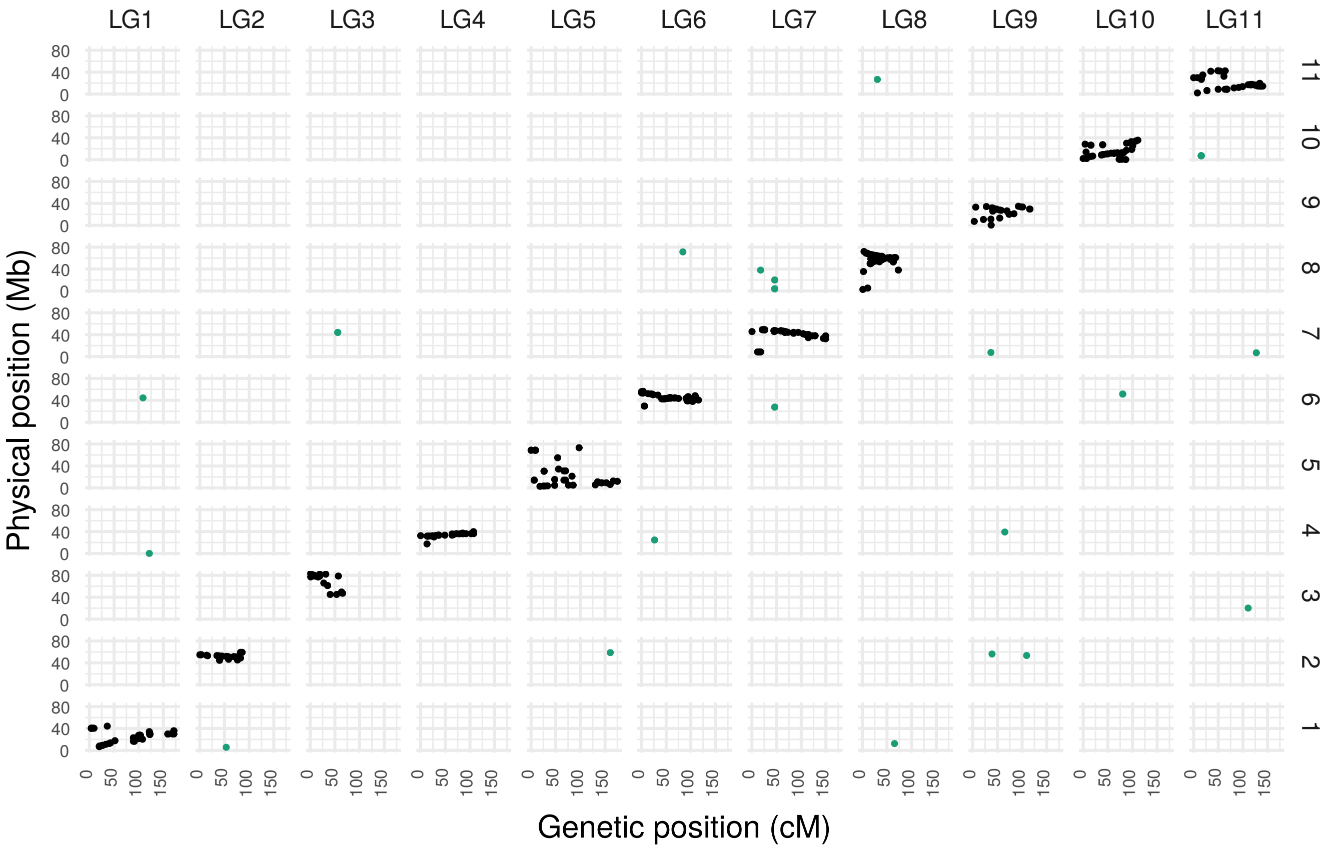

Figure S4. Comparison of composite genetic map of *Acca sellowiana* to *Eucalyptus grandis* genome sequence. The genetic position of Discovery-identified SNPs mapped in the *A. sellowiana* composite map was compared to physical position on *E. grandis* genome sequence. For each linkage group, SNPs corresponding to the reference chromosome of *E. grandis* are labeled in black. The green points represented SNPs in discordant reference chromosomes.

REFERENCES

Quezada, M., Pastina, M. M., Ravest, G., Silva, P., Vignale, B., Cabrera, D., et al. (2014). A first genetic map of *Acca sellowiana* based on ISSR, AFLP and SSR markers. *Sci. Hortic.* 169, 138–146. doi:doi.org/10.1016/j.scienta.2014.02.009
